# Multi-kinase inhibitors interact with sildenafil and ERBB1/2/4 inhibitors to kill tumor cells *in vitro* and *in vivo*

**DOI:** 10.18632/oncotarget.9752

**Published:** 2016-05-31

**Authors:** Laurence Booth, Thomas Albers, Jane L. Roberts, Mehrad Tavallai, Andrew Poklepovic, Iryna O. Lebedyeva, Paul Dent

**Affiliations:** ^1^ Department of Biochemistry and Molecular Biology, Virginia Commonwealth University, Richmond, VA 23298, USA; ^2^ Department of Medicine, Virginia Commonwealth University, Richmond, VA 23298, USA; ^3^ Department of Chemistry and Physics, Augusta University, Summerville Campus, Augusta GA 30912, USA

**Keywords:** sorafenib, pazopanib, chaperones, ERBB, PI3K

## Abstract

We have recently demonstrated that multi-kinase inhibitors such as sorafenib and pazopanib can suppress the detection of chaperones by *in situ* immuno-fluorescence, which is further enhanced by phosphodiesterase 5 inhibitors. Sorafenib and pazopanib inhibited the HSP90 ATPase activity with IC50 values of ~1.0 μM and ~75 nM, respectively. Pazopanib docked in silico with two possible poses into the HSP90 ATP binding pocket. Pazopanib and sildenafil combined to reduce the total protein levels of HSP1H/p105 and c-MYC and to reduce their co-localization. Sorafenib/pazopanib combined with sildenafil in a [GRP78+HSP27] –dependent fashion to: (i) profoundly activate an eIF2α/Beclin1 pathway; (ii) profoundly inactivate mTOR and increase ATG13 phosphorylation, collectively resulting in the formation of toxic autophagosomes. In a fresh PDX isolate of NSCLC combined knock down of [ERBB1+ERBB3] or use of the ERBB1/2/4 inhibitor afatinib altered cell morphology, enhanced ATG13 phosphorylation, inactivated NFκB, and further enhanced [sorafenib/pazopanib + sildenafil] lethality. Identical data to that with afatinib were obtained knocking down PI3K p110α/β or using buparlisib, copanlisib or the specific p110α inhibitor BYL719. Afatinib adapted NSCLC clones were resistant to buparlisib or copanlisib but were more sensitive than control clones to [sorafenib + sildenafil] or [pazopanib + sildenafil]. Lapatinib significantly enhanced the anti-tumor effect of [regorafenib + sildenafil] *in vivo*; afatinib and BYL719 enhanced the anti-tumor effects of [sorafenib + sildenafil] and [pazopanib] *in vivo*, respectively.

## INTRODUCTION

We have recently published that phosphodiesterase 5 inhibitors (sildenafil; tadalafil; vardenafil) can enhance the lethality of traditional cytotoxic chemotherapy agents as well as the lethality of the multi-kinase inhibitors sorafenib, pazopanib and regorafenib, and of celecoxib and a non-COX2 inhibitory analogue of the drug OSU-03012. Although activation of the death receptor CD95 could play a role in the drug interactions, other data argued that prolonged high levels of autophagosome formation also were essential for the lethal synergistic combination effects with the PDE5 inhibitor [1–4].

The drug OSU-03012 (AR12) was originally thought to act as an anti-cancer agent by inhibiting the enzyme PDK-1 within the PI3K pathway however it was subsequently shown that this compound was *not* primarily acting as a PDK-1 inhibitor, and subsequently it was demonstrated that the primary mechanism by which AR-12 killed tumor cells was via the PKR-like endoplasmic reticulum kinase (PERK) -dependent induction of endoplasmic reticulum stress signaling and a toxic form of autophagy. Other studies then linked the effects of AR-12 on tumor cell biology to the regulation of chaperone proteins [4, 5]. In more recent studies, we have shown that sorafenib, pazopanib, AR-12 and regorafenib can reduce the apparent expression chaperone proteins HSP90, GRP78 and HSP70 using an in-cell western/immuno-fluorescence approach [5–12]. As OSU-03012, sorafenib, pazopanib and regorafenib down-regulate the PERK inhibitory chaperone GRP78, and as the induction of toxic autophagy was PERK dependent, we investigated the role of reduced GRP78 expression caused by these drugs in the regulation of drug toxicity. We demonstrated that the drug OSU-03012 did not alter the transcription of GRP78 to any significant extent but instead destabilized the GRP78 protein itself, considerably reducing its half-life as assessed by western blotting from > 24 hours to approximately 10 hours. Over-expression of GRP78 prevented OSU-03012 induced PERK activation and eIF2α phosphorylation; autophagy induction, and tumor cell killing.

It is well-known that multiple chaperone proteins play essential roles in maintaining protein stability and cell signaling, most particularly in tumor cells which generally express much greater amounts of cellular protein than non-transformed cells. e.g. multiple myeloma cells [13, 14]. Thus some chaperone proteins, e.g. HSP90, have been the target for many developmental therapeutic chemists and also tumor cell biology researchers [15, 16]. Based on the fact our cancer biology data with chaperones and OSU-03012, sorafenib, pazopanib and regorafenib was congruent with the wider literature exploring the roles of chaperones in virus biology, we recently performed studies to determine whether these drugs could alter virus reproduction *in vitro* [7, 8]. In these studies, we discovered that OSU-03012, pazopanib or sorafenib all exhibited strong anti-viral properties against a wide range of DNA and RNA viruses [17]. Using molecular tools, we proved that the down-regulation of GRP78, HSP90, HSP70 and HSP27 was an essential property of both drugs in preventing virus reproduction.

The present oncology focused studies were initiated to determine whether sorafenib or pazopanib altered the expression/localization of additional chaperone proteins and to characterize their effects on chaperone and tumor cell biology.

## RESULTS

**Figure 1:**
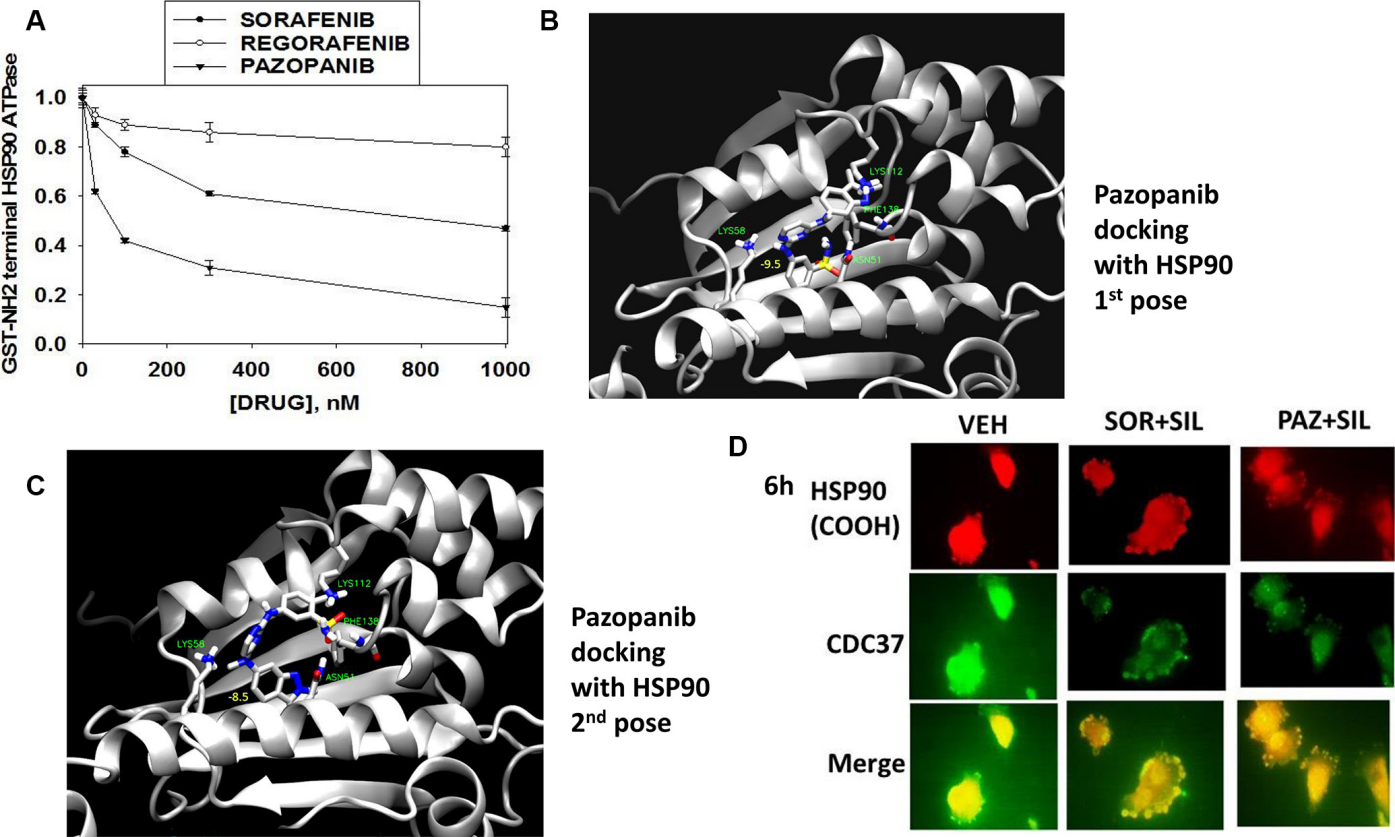
Sorafenib and pazopanib inhibit HSP90 ATPase activity; pazopanib docks with the ATP binding site of HSP90 (**A**) A GST fusion protein of the NH2-terminal fragment of HSP90 was synthesized in *E. coli* and purified on Glutathione Sepharose 4B beads. ATPase activity measurements were performed as described in the Methods in the presence of sorafenib, pazopanib or regorafenib (assays in triplicate +/−SEM, performed three times, one study is shown). (**B** and **C**) The model system for HSP90 was built using VMD. For the model, the coordinates of human HSP90 in complex with ACP (PDB refcode 3T10) were used. The protein was inserted into a cubic water box of size 80 × 80 × 80 Å^3^. The whole system consisted of 47971 atoms. For MD simulations, NAMD 2.10 was used. Ligand and protein were restrained to their original positions and the system was equilibrated at constant temperature and pressure (300 K, 1 atm, NPT ensemble) for 0.5 ns. Simulations were done using the CHARMM36 force-field in combination with the CGenFF forcefield where needed. Then 5 ns of production MD was performed. Docking (Autodock Vina) was done against conformations extracted from this run. Initial CGenFF-compatible parameters for the pazopanib were derived using the CGenFF website and subsequently optimized. Subsequently, 50 ns of accelerated MD with Generalized Born Implicit Solvent (GBIS) for both HSP90 in complex with pazopanib and apo-HSP90 was performed. (**D**) GBM12 cells were treated with vehicle, sorafenib (2.0 μM)/pazopanib (2.0 μM) and sildenafil (2.0 μM) for 6 h after which cells were fixed in place and permeabilized using 0.5% Triton X100. Immuno-fluorescence was performed to detect the expression levels of HSP90, CDC37 and their co-localization, using antibodies that recognize the HSP90 COOH-terminus epitope at 60× magnification.

We initially investigated whether sorafenib, pazopanib or regorafenib altered chaperone ATPase activity. We transformed bacteria with a plasmid to make a GST fusion protein of the NH_2_-terminal portion of HSP90; the domain that contains the ATP binding site and ATPase activity of the chaperone. Sorafenib and pazopanib, but not regorafenib, reduced chaperone ATPase activity, as measured on the isolated purified NH_2_-terminal HSP90-GST protein fragment *in vitro* (Figure 1A) see additional data in Booth et al, 2016: reference [9]. Based on chemical structure alone, the only difference between sorafenib and regorafenib is the inclusion of a single fluorine atom in regorafenib. It should be noted, however, that in our recent biochemical studies using mammalian cell synthesized HSP90 and HSP70; the PKG-dependent phosphorylation of these chaperones facilitated the ATPase inhibitory activity of regorafenib [9]. In silico docking of pazopanib into the amino-terminal ATP binding domain of HSP90 resulted in the identification of two predominant poses. In the first one, Asp51 makes a hydrogen bond with the sulfonamide oxygen, Lys58 makes a hydrogen bond with the pyrimidine nitrogen, and the amide hydrogen of Phe138 makes a hydrogen bond with the azaindole nitrogen. There is also a possible π-cation interaction between Lys112 and the five-membered ring of the azaindole (Figure 1B). In the second pose the ligand adopts the opposite orientation. Asn51 interacts with the indazole nitrogen, there is a possible π-cation interaction between Lys58 and the pyrimidine ring, and Lys112 and the amide hydrogen are creating hydrogen bonds with the sulfonamide group (Figure 1C). When combined with sildenafil both sorafenib and pazopanib modestly decreased HSP90 expression in 6 h as measured using a COOH terminal antibody but strongly reduced expression of its essential co-chaperone CDC37 that also correlated with reduced co-localization as judged by the pure-yellow merged signal becoming more orange in hue (Figure 1D) [9].

**Figure 2:**
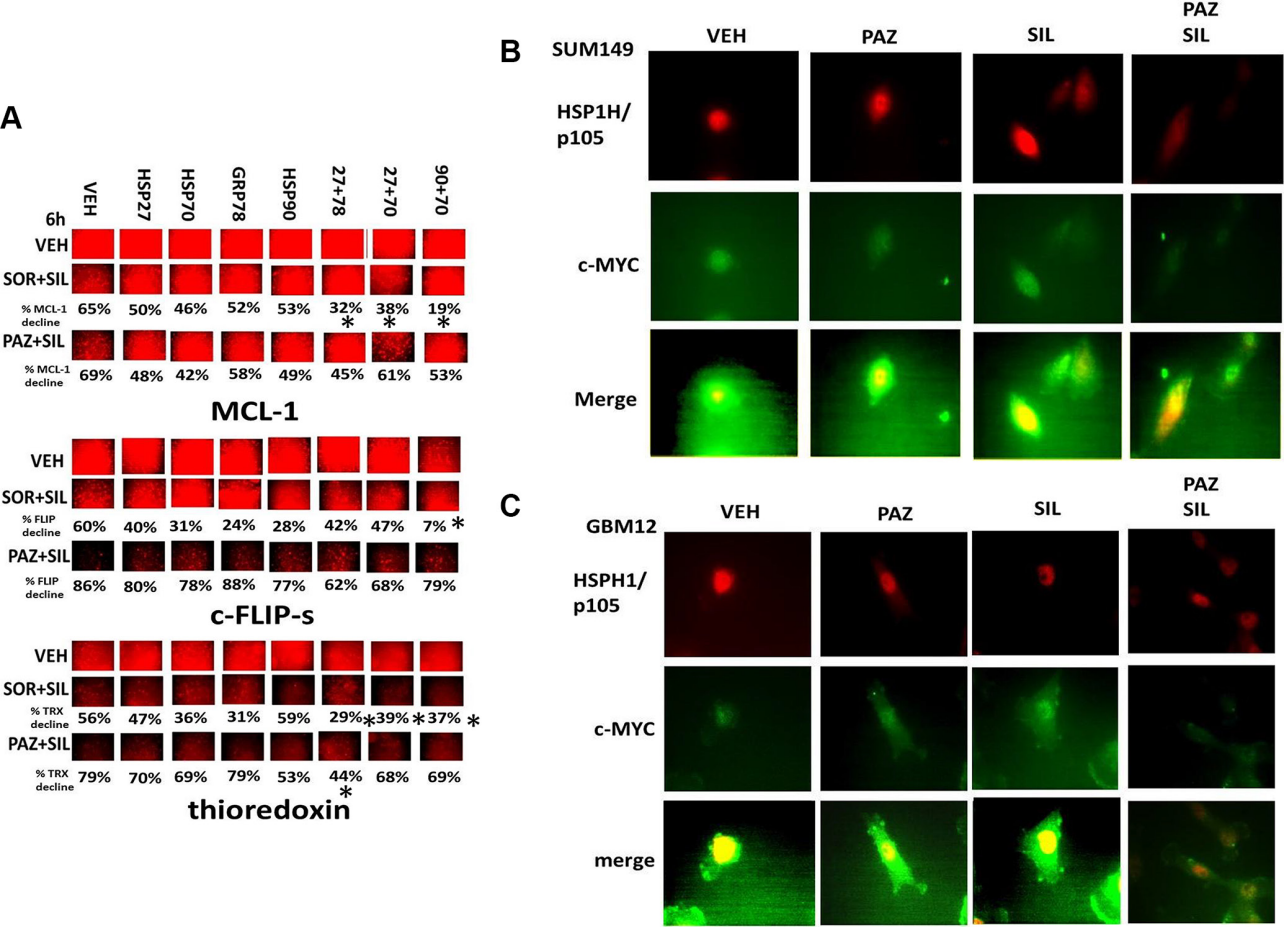
Assessing chaperone expression by immuno-fluorescence after sorafenib or pazopanib treatment (**A**) GBM12 cells were transfected with an empty vector plasmid or with plasmids to express HSP90, HSP70, GRP78, HSP27 or in the combinations indicated. Twenty four h after transfection cells were treated with vehicle control or with [sorafenib (2 μM) + sildenafil (2 μM)]; [pazopanib (2 μM) + sildenafil (2 μM)]. Six h after drug exposure, cells were fixed in place and permeabilized using 0.5% Triton X100. Immuno-fluorescence was performed to detect the expression levels of the indicated proteins (*p < 0.05 less reduction than in CMV control, *n* = 3, +/− SEM). (**B** and **C**) SUM149 and GBM12 cells, as indicated, were treated with vehicle control or with [pazopanib (2.0 μM) +/− sildenafil (2 μM)] for 6 h. After 6 h cells were then fixed in place and permeabilized using 0.5% Triton X100. Immuno-fluorescence was performed to detect the expression levels and the co-localization of HSPH1/p105 and of c-MYC. Images were at 60X magnification.

We then determined the impact of chaperone over-expression on the biologic actions of [sorafenib + sildenafil] and of [pazopanib + sildenafil]. Chaperone over-expression prevented either [sorafenib + sildenafil] or [pazopanib + sildenafil] from down-regulating MCL- 1 expression whereas the chaperones protected c-FLIP-s expression from only [sorafenib + sildenafil] (Figure 2A). Chaperone over-expression could not prevent either drug combination from reducing thioredoxin levels. The transcription factor c-MYC has for many decades been known as a potent mediator of the transformed/tumorigenic phenotype. Although c-MYC has been a considered a major target for anti-cancer drug development very little progress has been made at directly blocking c-MYC function. Indeed, a recent global UK-American initiative to develop therapies against cancer had listed as one area of research to be the inhibition of c-MYC. As with many proteins in the cell c-MYC associates with chaperones to maintain its tertiary structure and thereby function. Well-recognized chaperones for c-MYC include HSPH1/p105 – a member of the HSP70 superfamily, and HSP60, a chaperone we have previously shown to be inhibited by [pazopanib + sildenafil] [18, 19]. [Pazopanib + sildenafil] treatment reduced c-MYC expression and reduced both HSPH1/p105 and c-MYC co-localization concomitant with reduced the nuclear/peri-nuclear staining of c-MYC (Figures 2B and 2C).

**Figure 3:**
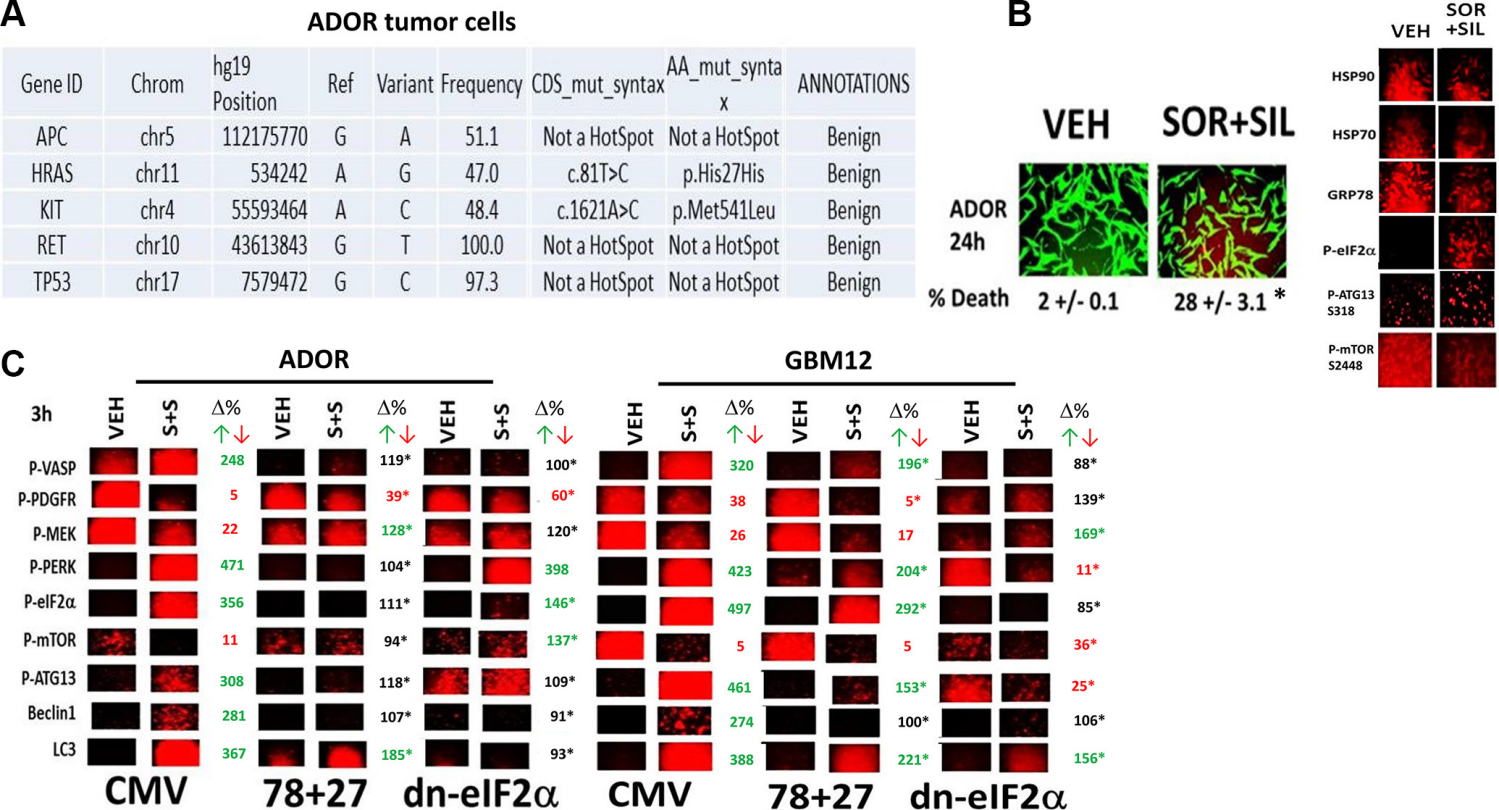
Over-expression of [GRP78 + HSP27] prevents [sorafenib + sildenafil] from causing endoplasmic reticulum stress signaling and prevents inactivation of mTOR and ATG13 phosphorylation (**A**) ADOR cells, a July 2015 PDX NSCLC isolate, were subjected to an Ion Ampli-Seq™ Cancer Hotspot Panel v2 screen for mutations in 50 genes, performed by the VCU Health System/Department of Pathology. (**B**) Left: ADOR cells, a July 2015 PDX NSCLC isolate, were treated with vehicle control or with [sorafenib (1.0 μM) + sildenafil (2 μM)] for 24 h after which cell viability was determined (*p < 0.05, *n* = 3 +/− SEM); Right: ADOR cells were treated with vehicle control or with [sorafenib (1.0 μM) + sildenafil (2 μM)] for 6 h. Cells were then fixed in place and permeabilized using 0.5% Triton X100. Immuno-fluorescence was performed to detect the expression levels of HSP90, GRP78 and HSP70, and the Serine 51 phosphorylation of eIF2α and Serine 318 phosphorylation of ATG13 and Serine 2448 phosphorylation of mTOR. (**C**) ADOR cells and GBM12 cells were transfected with empty vector plasmid, plasmids to over-express [HSP27 + GRP78], or a plasmid to express dominant negative eIF2α serine 51 to alanine. Twenty four h after transfection cells were treated with vehicle or [sorafenib (2.0 μM) and sildenafil (2.0 μM)] in combination for 3 h. Cells were fixed in place and permeabilized using 0.5% Triton X100. Immuno-fluorescence was performed to detect the expression levels of Beclin1 and LC3 and the phosphorylation levels of: VASP1; PDGFR; MEK1; PERK; eIF2α; mTOR; and ATG13. Values in red or green represent statistically significant changes in the fluorescence level compared to vehicle control, *p* < 0.05, *n* = 3 +/− SEM whereas values in black represent no statistically significant change; values labeled with an * are statistically different *p* < 0.05 from the parallel value in empty vector transfected cells.

In ADOR tumor cells, a July 2015 PDX model of non-small cell lung cancer that was fully localized within one lobe of the right lung, we discovered using genetic screening tools that these cells did not contain any “well described” mutated drivers for tumor growth, including “the guardian of the genome,” p53 (Figure 3A; 50 genes were tested, only 5 showed any genetic variation from the expected sequence). The combination of [sorafenib + sildenafil] killed ADOR cells that correlated with reduced expression of HSP90, HSP70 and GRP78 using NH_2_-terminal antibodies for detection; with reduced phosphorylation of mTOR S2448; and with increased phosphorylation of eIF2α S51 and ATG13 S318 (Figure 3B).

Based on the present studies, in addition to those in Tavallai et al and Roberts et al, we next explored how [sorafenib + sildenafil] treatment was regulating the phosphorylation and expression of biomarkers and autophagy regulatory proteins. In ADOR cells we noted, 3 h after treatment at 10X magnification to detect gross changes in the fluorescence signal, that [sorafenib + sildenafil] treatment: increased VASP phosphorylation – a PKG activity marker; decreased PDGFR and MEK phosphorylation – sorafenib effect markers; increased PERK and eIF2α phosphorylation and elevated Beclin1 and LC3 expression – endoplasmic reticulum stress markers for GRP78 down-regulation and increased autophagosome formation; decreased mTOR phosphorylation and elevated ATG13 phosphorylation – markers for increased autophagosome formation (Figure 3C). In both ADOR and GBM12 cells, to our surprise, over-expression of GRP78 and HSP27 or expression of dominant negative eIF2α S51A suppressed VASP phosphorylation, suggesting that the ability of sildenafil to activate PKG has been impaired. Over-expression of GRP78 and HSP27 suppressed the drug –induced phosphorylation of PERK, eIF2α and ATG13, prevented dephosphorylation of mTOR, and suppressed the expression of Beclin1 and LC3. Expression of dominant negative eIF2α S51A suppressed PERK-eIF2α phosphorylation, prevented the inactivation of mTOR, and prevented the drug combination from increasing Beclin1 and LC3 expression. To our surprise, in both cell types, expression of eIF2α S51A increased basal levels of ATG13 S318 phosphorylation; and as basal levels of mTOR phosphorylation either remained unchanged or fell when eIF2α S51A was expressed, this argues that protein phosphatase activity against ATG13 S318 must have declined. Control data showing GRP78 and HSP27 knock downs and eIF2α over-expression is presented in Figure S1A.

**Figure 4:**
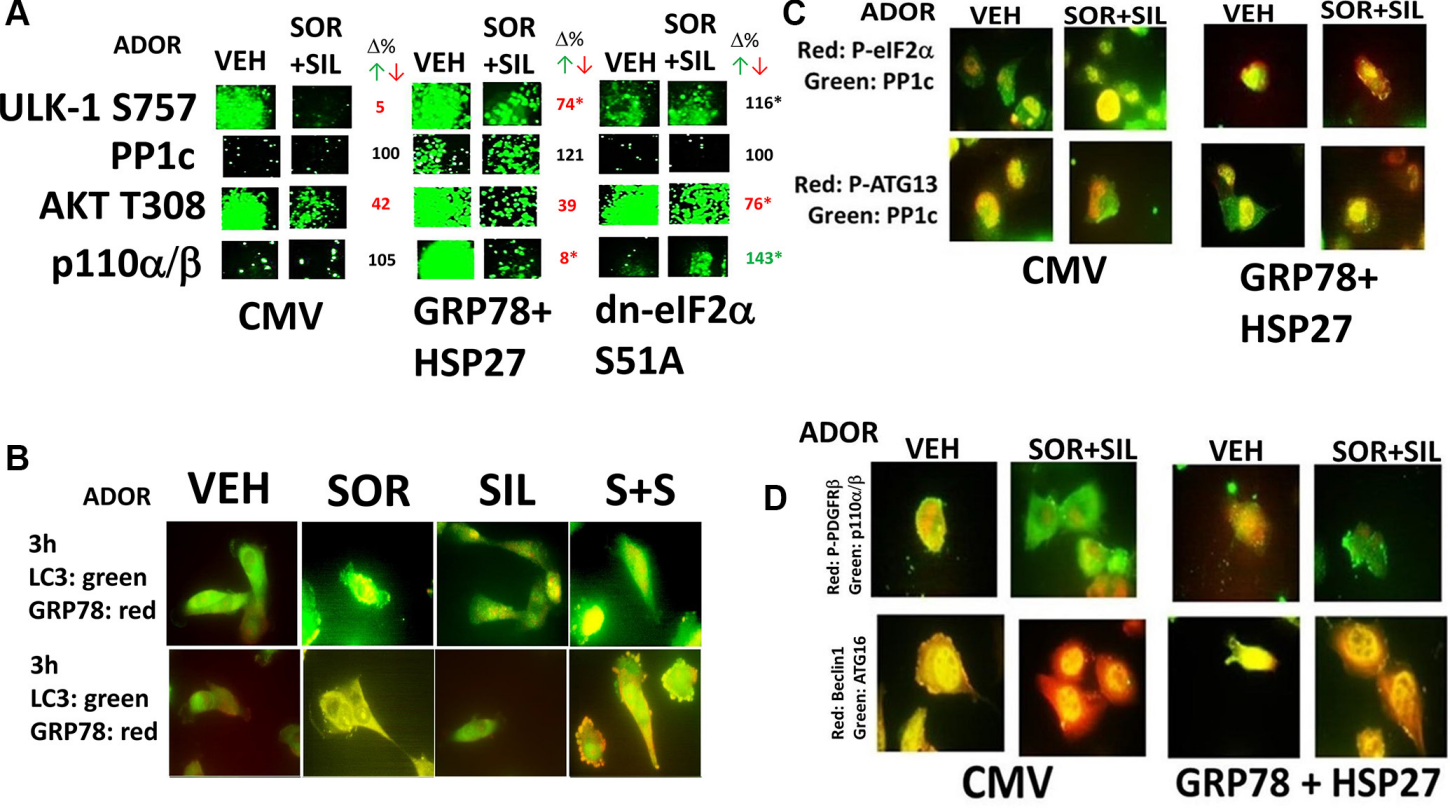
[Sorafenib + sildenafil] regulate the phosphorylation levels of autophagy regulatory proteins (**A**) ADOR cells were transfected with empty vector plasmid, plasmids to over-express [HSP27 + GRP78], or a plasmid to express dominant negative eIF2α serine 51 to alanine. Twenty four h after transfection cells were treated with vehicle or [sorafenib (2.0 μM) and sildenafil (2 μM)] for 3 h after which cells were fixed in place and permeabilized using 0.5% Triton X100. Immuno-fluorescence was performed to detect the expression/phosphorylation levels of: ULK-1 S757; PP1c; AKT T308; PI3K p110α/β. Values in red or green represent statistically significant changes in the fluorescence level compared to vehicle control, *p* < 0.05, *n* = 3 +/− SEM whereas values in black represent no statistically significant change; values labeled with an * are statistically different *p* < 0.05 from the parallel value in empty vector transfected cells. (**B**) ADOR cells were treated with vehicle, sorafenib (2.0 μM) and/or sildenafil (2 μM) for 3 h. Cells were fixed in place and permeabilized using 0.5% Triton X100. Immuno-fluorescence was performed at 60× magnification to detect the co-localization of HSP27 with LC3 and of GRP78 and LC3. (**C**) ADOR cells were transfected with empty vector plasmid, or plasmids to over-express [HSP27 + GRP78]. Twenty four h after transfection cells were treated with vehicle or [sorafenib (2.0 μM) and sildenafil (2 μM)] for 3 h after which cells were fixed in place and permeabilized using 0.5% Triton X100. Immuno-fluorescence at 60× was performed to detect the co-localization levels of: PP1c and phospho-eIF2α; PP1c and phospho-ATG13. (**D**) ADOR cells were transfected with empty vector plasmid, or plasmids to over-express [HSP27 + GRP78]. Twenty four h after transfection cells were treated with vehicle or [sorafenib (2.0 μM) and sildenafil (2 μM)] for 3 h after which cells were fixed in place and permeabilized using 0.5% Triton X100. Immuno-fluorescence at 60X was performed to detect the co-localization levels of: Beclin1 and ATG16; and phospho-PDGFRα/β and PI3K p110α/β.

The protein kinase ULK-1 is considered to be an essential gate-keeper kinase for the regulation of autophagosome formation, and whose activity is negatively regulated by Serine 757 phosphorylation, catalyzed by mTOR [9, 11, 12]. The reduced mTOR S2448 phosphorylation caused by drug exposure and shown in Figure 3 correlated with the drug-induced dephosphorylation of ULK-1 at Serine 757, which collectively in turn correlated with increased phosphorylation of the autophagosome formation regulatory protein ATG13 at Serine 318 seen in Figure 3 (Figure 4A) [20, 21]. In cells over-expressing [GRP78 + HSP27] phosphorylation of Serine 757 was modestly elevated under basal conditions and in cells expressing eIF2α S51A, the phosphorylation of Serine 757 under basal conditions was lower compared to control transfected cells. Over-expression of [GRP78 + HSP27] or expression of eIF2α S51A impeded the ability of the drug combination to reduce ULK-1 S757 phosphorylation. Treatment of cells with [sorafenib + sildenafil] reduced AKT T308 phosphorylation, an effect that was partially reduced by over-expression of [GRP78 + HSP27] or in cells expressing eIF2α S51A. Over-expression of [GRP78 + HSP27] increased the basal expression levels of PI3K p110α/β, that is in agreement with data of others arguing that these chaperones facilitate PI3K pathway signaling [22, 23].

Studies in Tavallai et al demonstrated that induction of a toxic form of autophagy plays a key central role in [sorafenib + sildenafil] lethality [6]. Treatment of cells with sorafenib caused a modest co-localization of LC3 and GRP78 as judged by formation of punctate yellow vesicles (Figure 4B, upper). [Sorafenib + sildenafil] caused large dense areas of yellow staining. Sorafenib as a single agent also caused LC3 and HSP27 to co-localize though no obvious small punctate structures had formed (Figure 4B, lower). Treatment with [sorafenib + sildenafil] caused considerable vesicularization with the vesicles having orange/red staining, suggestive that either HSP27 had been broken down in the vesicles or had dissociated from LC3 and the vesicles.

In control vector transfected cells [sorafenib + sildenafil] treatment decreased the co-localization of PP1c with phospho-ATG13 and increased the co-localization of PP1c with phospho-eIF2α. These co-localization observations were blocked by over-expression of [GRP78 + HSP27] or by expression of eIF2α S51A (Figure 4C, not shown). Treatment of cells with [sorafenib + sildenafil] reduced the co-localization of phospho-PDGFR with PI3K p110α/p110β (Figure 4D, not shown). Beclin1 and ATG16 co-localized under all conditions with the composition of the co-localization varying slightly between drug treatment or transfection condition. Collectively these data argue that [sorafenib + sildenafil] treatment promotes autophagosome formation in cancer cells through multiple inter-locking mechanisms: decreased chaperone function resulting in elevated ATG13 phosphorylation and ER stress –stimulated expression of Beclin1 and ATG13; and altered PP1c association with eIF2α and ATG13 which acts to maintain these signals.

**Figure 5:**
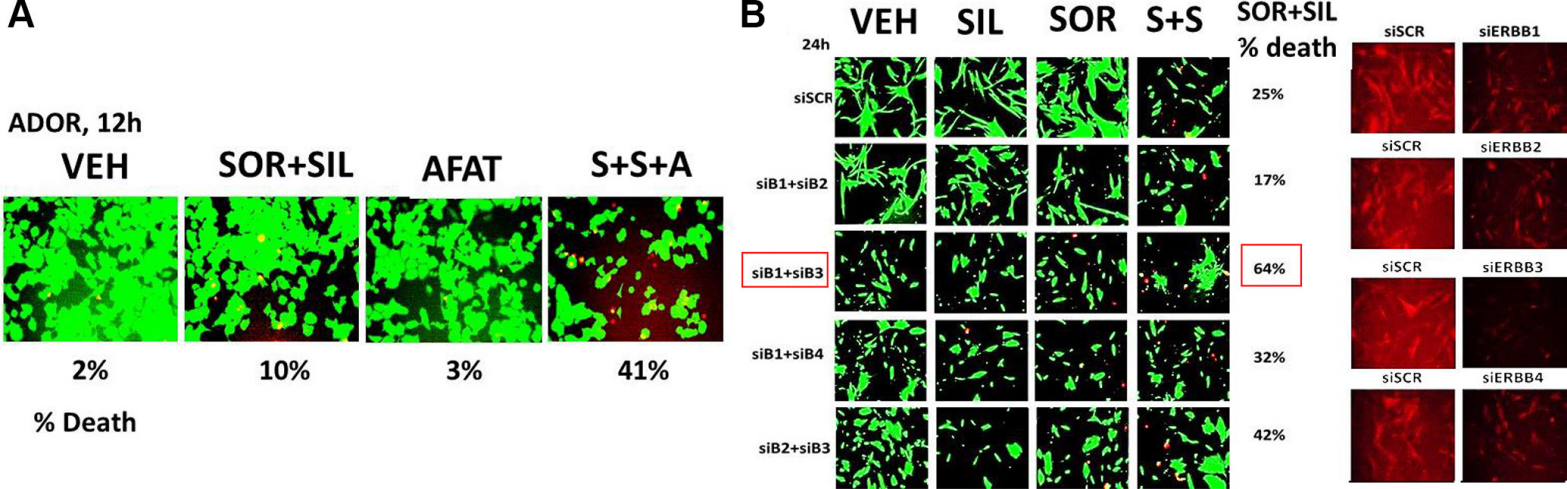
The siRNA knock down of ERBB1 and ERBB3 enhances [sorafenib + sildenafil] lethality against a freshly isolated PDX model of non-small cell lung cancer (**A**) ADOR cells were treated with vehicle control or with [sorafenib (2.0 μM) + sildenafil (2 μM)] in the presence or absence of afatinib (1 μM) for 24 h after which cell viability was determined (*n* = 3 +/− SEM). (**B**) ADOR cells as indicated were transfected with a scrambled siRNA or siRNA molecules alone or in combination to knock down expression of ERBB1, ERBB2, ERBB3 and ERBB4. Twenty four h after transfection cells were treated with vehicle control or with [sorafenib (2.0 μM) + sildenafil (2 μM)] for 24 h after which cell viability was determined (*n* = 3 +/− SEM).

The EGF-R (epidermal growth factor receptor), also known as HER-1, ErbB1, or ErbB, is a transmembrane receptor tyrosine kinase that binds a subset of the EGF family ligands [24, 25]. Our recent work has demonstrated using multiplexed antibody array detection approaches that surviving tumors previously treated *in vivo* with [sorafenib/regorafenib + sildenafil] exhibited activation of an ERBB1-PI3K-NFκB pathway [6]. Inhibition of ERBB1/2/4 signaling using the second-generation suicide inhibitor afatinib significantly enhanced [sorafenib + sildenafil] lethality (Figure 5A). Combined knock down of ERBB1 and ERBB3, but not individual knock down of either receptor, profoundly enhanced the lethality of [sorafenib + sildenafil] in ADOR cells (Figure 5B, *p* < 0.05). Similar data were obtained examining a wide range of other tumor cell types (Figure S1B). During these studies we discovered in vehicle control treated cells that combined knock down of ERBB1 and ERBB3 profoundly changed the morphology of ADOR cells, with cells lacking ERBB1 and ERBB3 exhibiting a more rounded and/or elongated appearance versus control cells that had a spindle-like appearance with multiple elongated thin projections; projections that are similar in morphology to neurite projections (Figure S1C). In ADOR cells a 6 h treatment with the second generation ERBB1/2/4 inhibitor drug afatinib also profoundly altered cell morphology which was associated with a modest reduction in the expression of the RTK-actin cytoskeleton regulatory protein WASF3 (Figure S1C). Afatinib treatment did not alter the expression level of tubulin or the phosphorylation of SRC Y416 or SYK Y525/Y526 but did increase SYK Y323 phosphorylation which is indicative of SYK inactivation (Figure S1D).

**Figure 6:**
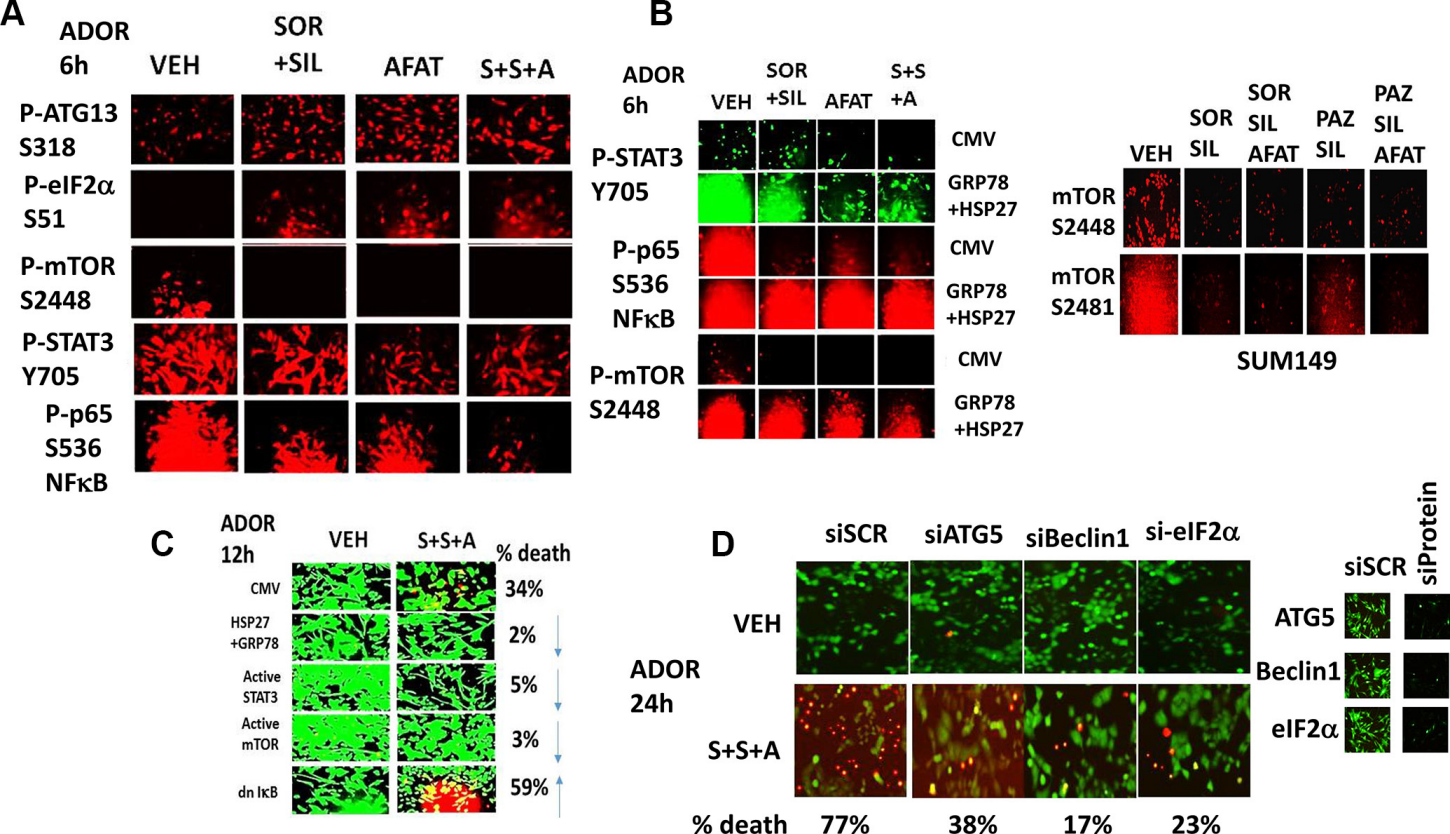
[Sorafenib + Sildenafil + Afatinib] treatment inactivates mTOR, STAT3 and NFκB; over-expression of [GRP78 + HSP27] or knock down of Beclin1 / ATG5 / eIF2α prevents these effects (**A**) ADOR cells were treated with vehicle control or [sorafenib (2.0 μM) + sildenafil (2 μM)] and/or afatinib (1 μM) or the three drugs in combination, for 6 h after which the phosphorylation of eIF2α, mTOR, ATG13, ERK1/2, AKT (T308), STAT3 (Y705) and p65 NFκB was determined at 10× by immuno-fluorescence in the Hermes WiScan system. (**B**) ADOR cells were transfected with an empty vector plasmid, or plasmids to express HSP27 and GRP78 together. Twenty four h after transfection cells were treated with vehicle control or with [sorafenib (2.0 μM) + sildenafil (2 μM)] +/− afatinib (1 μM) for 6 h after which cells were fixed in place and permeabilized using 0.5% Triton X100. Immuno-fluorescence was performed to detect the phosphorylation levels of mTOR, STAT3 and p65 NFκB. (**C**) ADOR cells were transfected with an empty vector plasmid, or plasmids to express: GRP78 and HSP27 together; an activated form of STAT3; an activated form of mTOR; or the dominant negative super-repressor IκB S32A S36A. Twenty four h after transfection cells were treated with vehicle control or with [sorafenib (2.0 μM) + sildenafil (2 μM) + afatinib (1 μM)] for 12 h after which cell viability was determined (n = 3 +/− SEM). (D) ADOR cells were transfected with a scrambled siRNA or siRNA molecules to knock down Beclin1 or ATG5 or eIF2α. Twenty four h after transfection cells were treated with vehicle control or with [sorafenib (2.0 μM) + sildenafil (2 μM) + afatinib (1 μM)] for 12 h after which cell viability wasdetermined (*n* = 3 +/−SEM).

In ADOR cells afatinib enhanced the ability of [sorafenib + sildenafil] treatment to decrease the phosphorylation of p65 NFκB S536 (Figure 6A). Of note, treatment with all three drugs increased the phosphorylation of eIF2α S51 and ATG13 S318 and decreased the phosphorylation of mTOR S2448 and STAT3 Y705. Combined over-expression of HSP27 and GRP78 prevented the drug-induced inactivation of mTOR, STAT3 and NFκB (Figure 6B). Of further note, the phosphorylation of mTOR at both Serine 2448 and Serine 2481 was reduced by the drug combination arguing that the TORC1 and the TORC2 complexes were both inactivated. Expression of an activated form of mTOR or an activated form of STAT3 in ADOR cells suppressed the lethality of [sorafenib + sildenafil + afatinib] treatment, and expression of the super-repressor IκB S32A S36A (Figure 6C, each *p* < 0.05). Knock down of Beclin1 or ATG5 or eIF2α expression significantly reduced the ability of [sorafenib + sildenafil + afatinib] treatment to kill tumor cells (Figure 6D).

**Figure 7:**
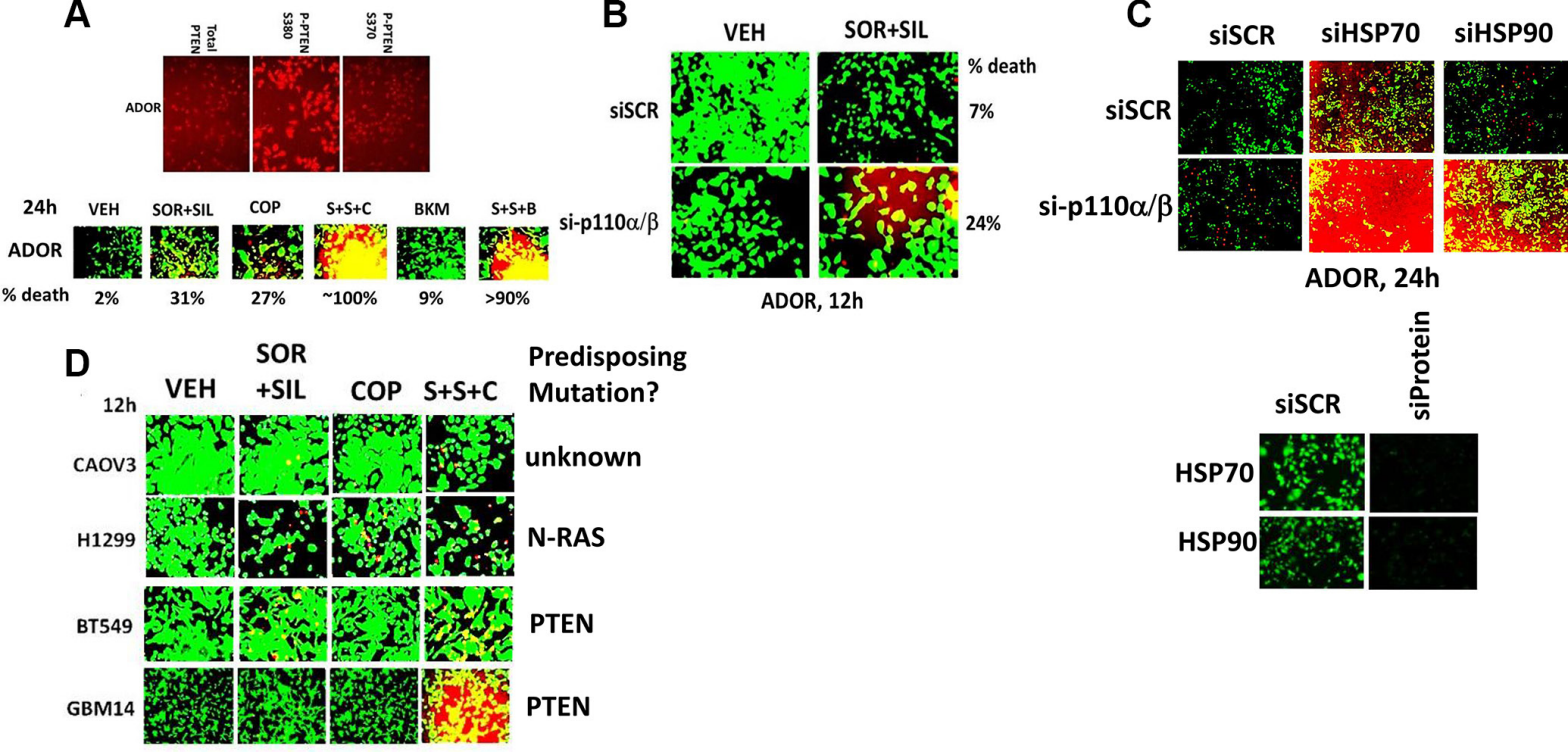
Inhibition of PI3K signaling enhances [sorafenib + sildenafil] lethality (**A**) Upper. ADOR cells, 24 h after plating, were fixed in place and permeabilized using 0.5% Triton X100. Immuno-fluorescence was performed to detect the expression level of PTEN and the phosphorylation of PTEN S380 and PTEN S370. Lower. ADOR cells were treated with vehicle control or with [sorafenib (2.0 μM) + sildenafil (2 μM)] +/− copanlisib (0.5 μM) +/− buparlisib (0.5 μM) for 12 h after which cell viability was determined (*n* = 3 +/− SEM). (**B**) ADOR cells were transfected with a scrambled siRNA or siRNA molecules to knock down the expression of both p110α and p110β. Twenty four h after transfection cells were treated with vehicle control or with [sorafenib (2.0 μM) + sildenafil (2 μM)] for 12 h after which cell viability was determined (*n* = 3 +/− SEM). (**C**) ADOR cells were transfected with a scrambled siRNA or siRNA molecules to knock down the expression of both p110α and p110β and/or in parallel transfected with scrambled siRNA or siRNA molecules to knock down the expression of both HSP90 and HSP70. Twenty four h after transfection cells were processed for cell viability assays (*n* = 3 +/− SEM). (**D**) Various tumor cells were treated with vehicle control or with [sorafenib (2.0 μM) + sildenafil (2 μM)] +/− copanlisib (0.5 μM)) for 12 h after which cell viability was determined (*n* = 3 +/− SEM).

In parallel to these studies we also examined expression of the tumor suppressor PTEN and also PTEN S380 phosphorylation in the ADOR isolate. Although PTEN was expressed in ADOR cells the ratio of phospho-S380 to total PTEN protein was 2.86, suggesting that in ADOR cells although the cells express a wild type PTEN, all of the wild type PTEN protein may be *de facto* inactivated by S380 phosphorylation (Figure 7A, upper). Inhibition of PI3K signaling using copanlisib (COP) or using buparlisib (BKM) profoundly enhanced [sorafenib + sildenafil] lethality (Figure 7A, lower). Molecular knock down of the p110α/β subunits of PI3K, significantly enhanced [sorafenib + sildenafil] killing (Figure 7B). Knock down of HSP70 and to a lesser extent HSP90 interacted in a greater than additive fashion with knock down of the PI3K p110α/β subunits to kill ADOR tumor cells (Figure 7C). Similar data were obtained in other tumor types including breast, brain, lung and ovarian cancer (Figure 7D).

As has been described in several contemporaneous studies from our group, we generated in the flanks of athymic nu/nu mice tumors of the double mutant active ERBB1 expressing lung cancer line H1975. Tumors were formed in animals, then the animals were treated with afatinib BID; all of the tumors completely disappeared. After ~1 week tumors slowly began to re-grow. From five tumors we isolated five afatinib resistant clones from each tumor, as judged being able to rapidly proliferate *in vitro* in the presence of 1 μM afatinib, for further *in vitro* study. Afatinib resistant H1975 clones exhibited higher levels of AKT T308 phosphorylation and lower levels of PTEN protein as well as PTEN S380 phosphorylation (Figure S2A). Expression of the negative transcriptional regulator of the PTEN gene, NEDD4, were much higher than in control clones, and that the expression of another NEDD4 target and PTEN target, PTPN13, were also low. PTPN13 (FAP-1) is a protein tyrosine phosphatase that is localized in the plasma membrane environment by PTEN, and reduced PTPN13 expression in afatinib resistant H1975 cells correlated with elevated tyrosine phosphorylation of ERBB3, PTEN Y315 and SRC Y416 [26]. We discovered that treatment with [sorafenib + sildenafil + copanlisib] was an effective modality at killing H1975 control cells, and killed the afatinib resistant H1975 clones to a greater extent than control clones (Figure S2B, *p* < 0.05).

**Figure 8:**
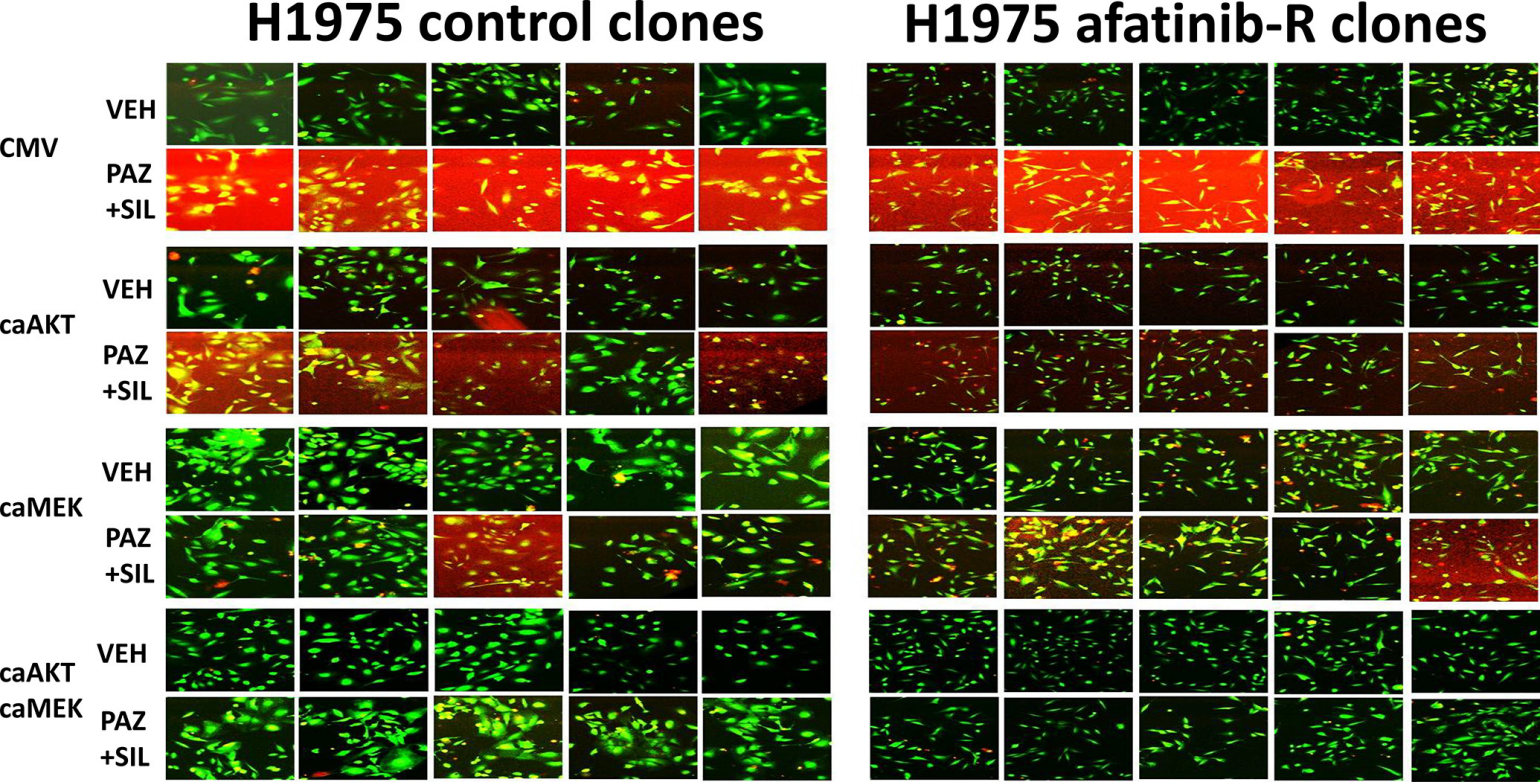
*In vivo* generated afatinib resistant H1975 clones are killed by [pazopanib + sildenafil] and are protected by dual, but not individual, activation of MEK1 and AKT signaling Control and afatinib resistant H1975 non-small cell lung cancer clones were transfected with an empty vector plasmid (CMV); a plasmid to express activated AKT; a plasmid to express activated MEK1; or with plasmids to express both activated AKT and activated MEK1. Twenty four h after transfection cells were treated with vehicle control or with [pazopanib (2.0 μM) + sildenafil (2 μM)] for 24 h as indicated, after which cell viability was determined in an Hermes WiScan microscope at 10× magnification. Cells staining red/yellow are dead; those staining green are viable (*n* = 3 +/− SEM).

**Figure 9:**
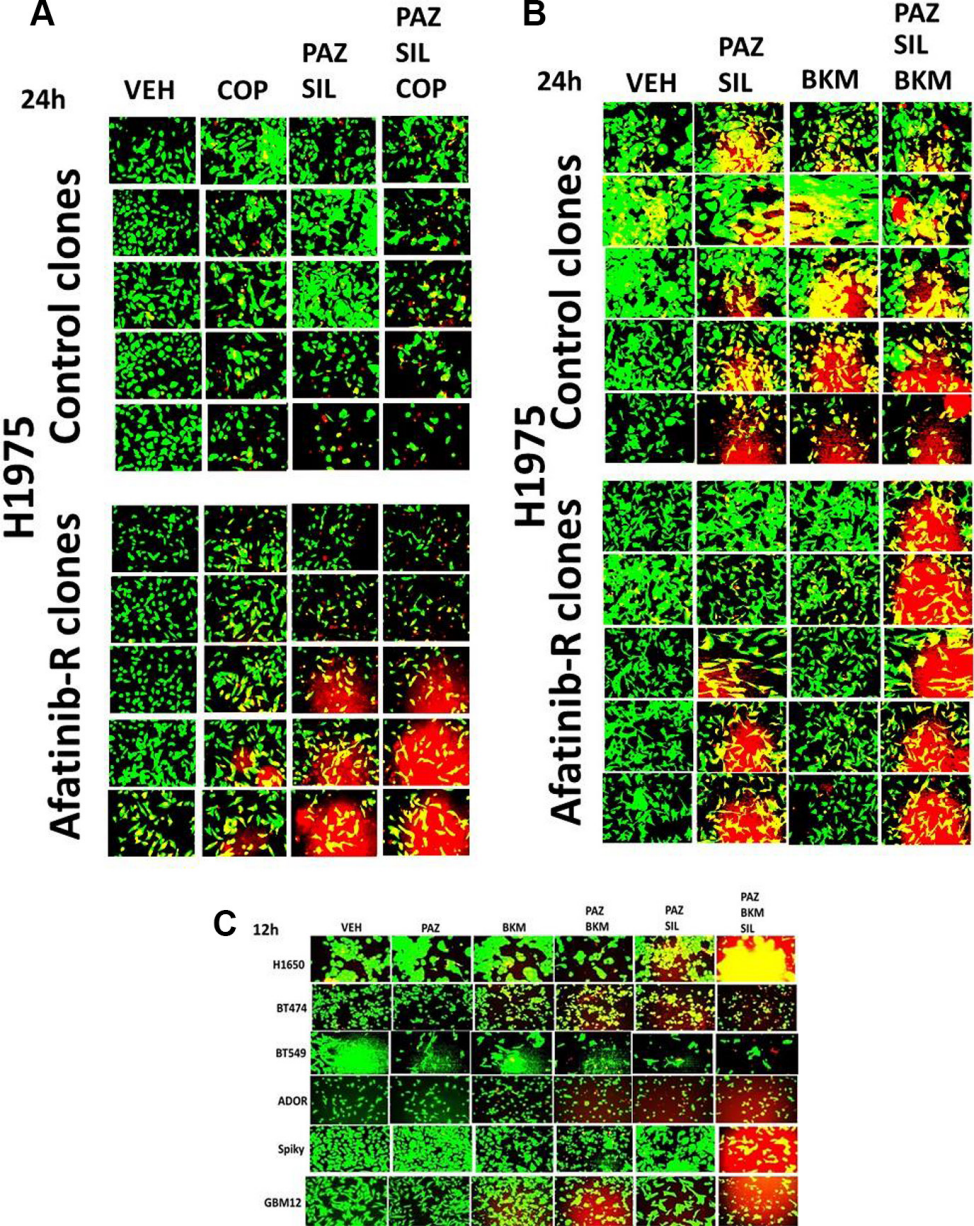
Pazopanib is as effective a drug as sorafenib when combined with sildenafil and PI3K inhibitors to kill cancer cells (**A–B**) Control and afatinib resistant H1975 clones were treated with vehicle control or with [pazopanib (2.0 μM) + sildenafil (2 μM)] +/− copanlisib (0.5 μM) or BKM120 (buparlisib) (0.5 μM) for 12 h after which cell viability was determined. (**C**) NSCLC cells (H1650, ADOR); breast cancer cells (BT474, BT549); brain cancer cells (GBM12); and ovarian cancer cells (Spiky) were treated with vehicle control or with [pazopanib (2.0 μM) + sildenafil (2 μM)] +/− buparlisib (BKM120) (0.5 μM) for 12 h after which cell viability was determined.

**Figure 10:**
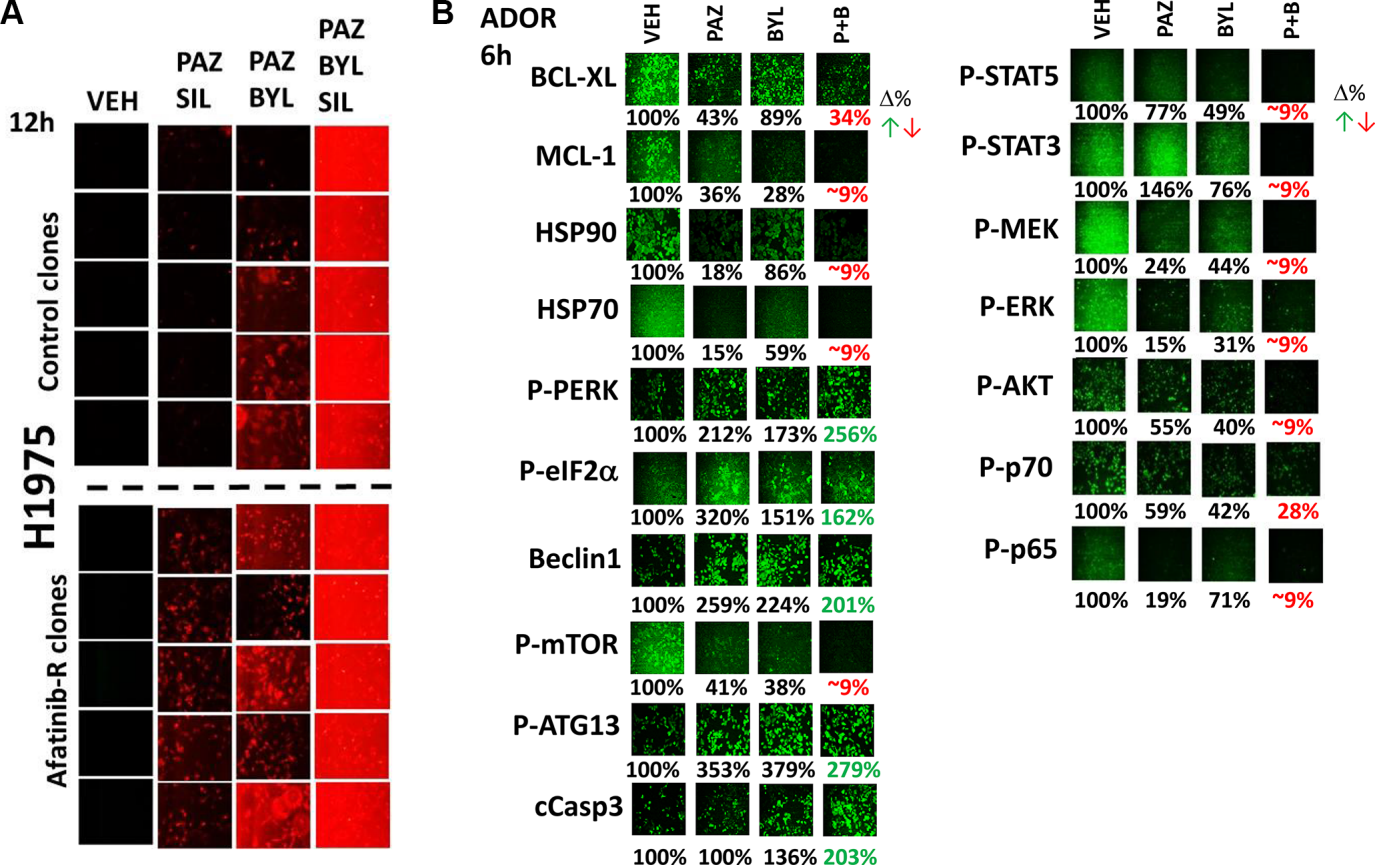
Pazopanib is as effective a drug as sorafenib when combined with sildenafil and PI3K inhibitors to kill a diverse range of cancer cell types (**A**) H1975 cells, as indicated in the Figure, were treated with vehicle control or with [pazopanib (2.0 μM) + sildenafil (2 μM)] +/− BYL719 (0.5 μM) for 12 h after which cell viability was determined. (**B**) ADOR cells were treated with vehicle control or with [pazopanib (2.0 μM) +/− BYL719 (0.5 μM)] for 6 h after which cells were fixed in place, permeabilized and immuno-fluorescence assays were performed to detect the indicated proteins and phosphorylation status of proteins at 10× magnification in the Hermes WiScan system. Values in red or green represent statistically significant changes in the fluorescence level compared to vehicle control, *p* < 0.05, *n* = 3 +/− SEM.

**Figure 11:**
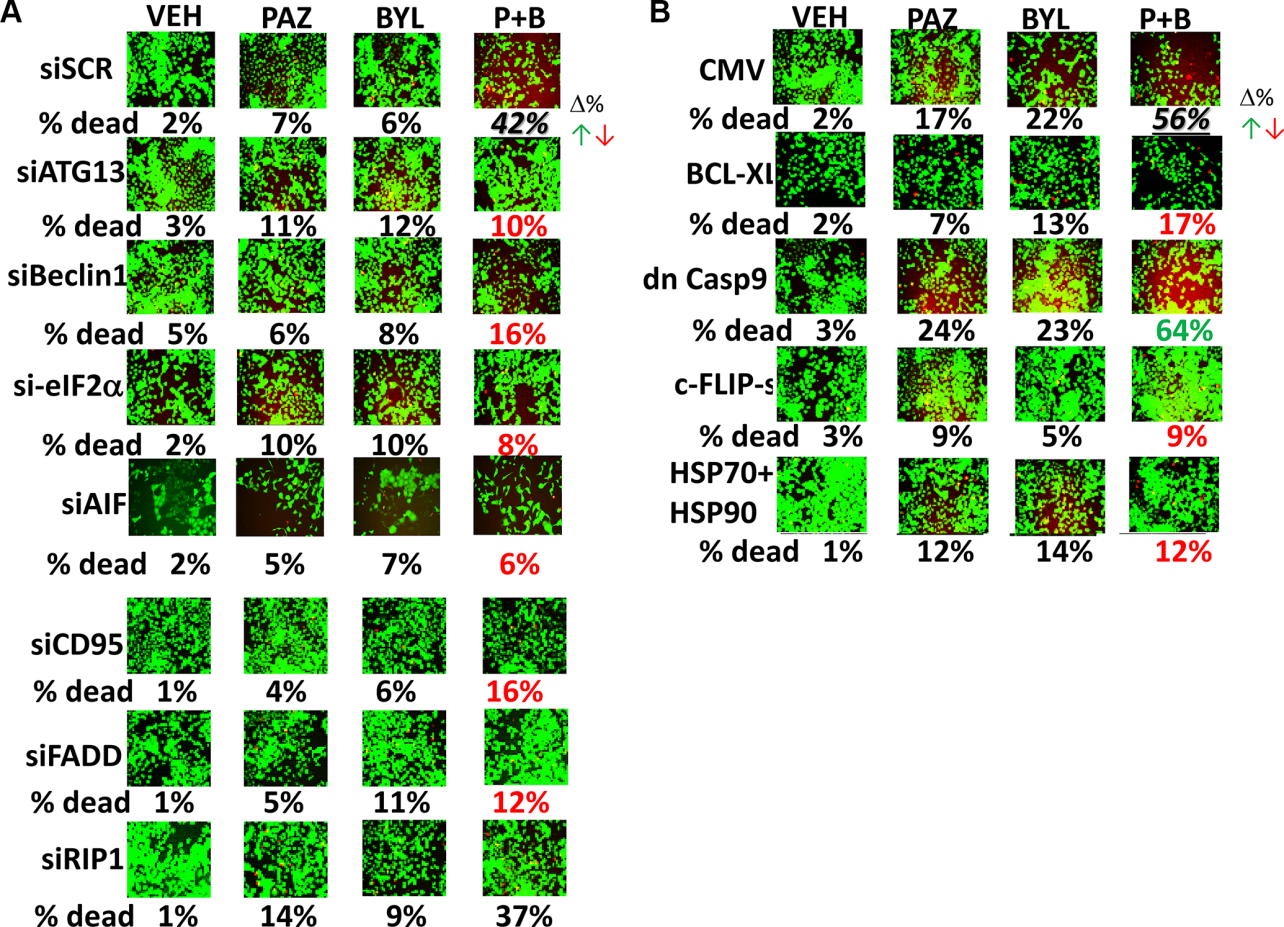
[Pazopanib + BYL719] kills NSCLC cells via an ER stress/autophagy dependent pathway that utilizes CD95-BCL-XL-AIF –dependent killing (**A**) ADOR cells were transfected with a scrambled siRNA or with validated siRNA molecules to knock down: ATG13; Beclin1; eIF2α; AIF; CD95; FADD; and RIP-1. Twenty-four h after transfection the cells were treated with vehicle control, pazopanib (2.0 μM), BYL719 (0.5 μM) or the drugs combined for 24 h after which cell viability was determined. Values in red or green represent statistically significant changes in the fluorescence level compared to vehicle control, *p* < 0.05, *n* = 3 +/− SEM. (**B**) ADOR cells were transfected with an empty vector plasmid (CMV) or with plasmids to express: BCL-XL; dominant negative caspase 9; c-FLIP-s; and [HSP90 + HSP70] combined. Twenty four h after transfection the cells were treated with vehicle control, pazopanib (2.0 μM), BYL719 (0.5 μM) or the drugs combined for 24 h after which cell viability was determined. Values in red or green represent statistically significant changes in the fluorescence level compared to vehicle control, *p* < 0.05, *n* = 3 +/− SEM.

Pazopanib and sildenafil interacted to kill control and afatinib resistant H1975 clones (Figure 8). In both control and afatinib resistant clones, activation of MEK-ERK1/2 was more protective than activation of AKT against [pazopanib + sildenafil] (Figure 8, *p* < 0.05). However, it was only combined activation of both MEK-ERK1/2 and AKT signaling that fully prevented drug combination lethality in all clones. The drug combination of [pazopanib + sildenafil] was as toxic to the H1975 clones as was [sorafenib + sildenafil], and the combinations of [pazopanib + sildenafil + buparlisib] and [pazopanib + sildenafil + copanlisib] caused levels of killing comparable to those seen using sorafenib (Figures 9A and 9B). Similar drug combination data were observed in multiple other tumor cell types, including PDX tumor models such as ADOR (lung); Spiky (ovarian); and GBM12 (glioblastoma) (Figure 9C) [27]. Based on the data in Figure 8, we next employed a PI3K inhibitor to combine with pazopanib. Pazopanib and the PI3K p110α/β specific inhibitor BYL719 weakly interacted to kill control H1975 clones, with afatinib resistant clones being significantly more sensitive to the combination than control clones (Figure 10A, *p* < 0.05). The addition of sildenafil to the [pazopanib + BYL719] combination caused a profound high level of tumor cell death with ~100% of the cells being killed within 24 h. The observed changes in cell viability correlated with decreased expression of cyto-protective proteins and cyto-protective signaling pathways and increased phosphorylation of proteins that signal for ER stress and autophagy (Figure 10B). Molecular inhibition of autophagy or ER stress signaling protected tumor cells from [pazopanib + BYL719] toxicity (Figure 11A; Figure S1A). Over-expression of HSP70 and HSP90; over-expression of c-FLIP-s or BCL-XL; or knock down of AIF, CD95 or FADD protected cells from [pazopanib + BYL719] exposure (Figure 11B; Figure S1A).

**Figure 12:**
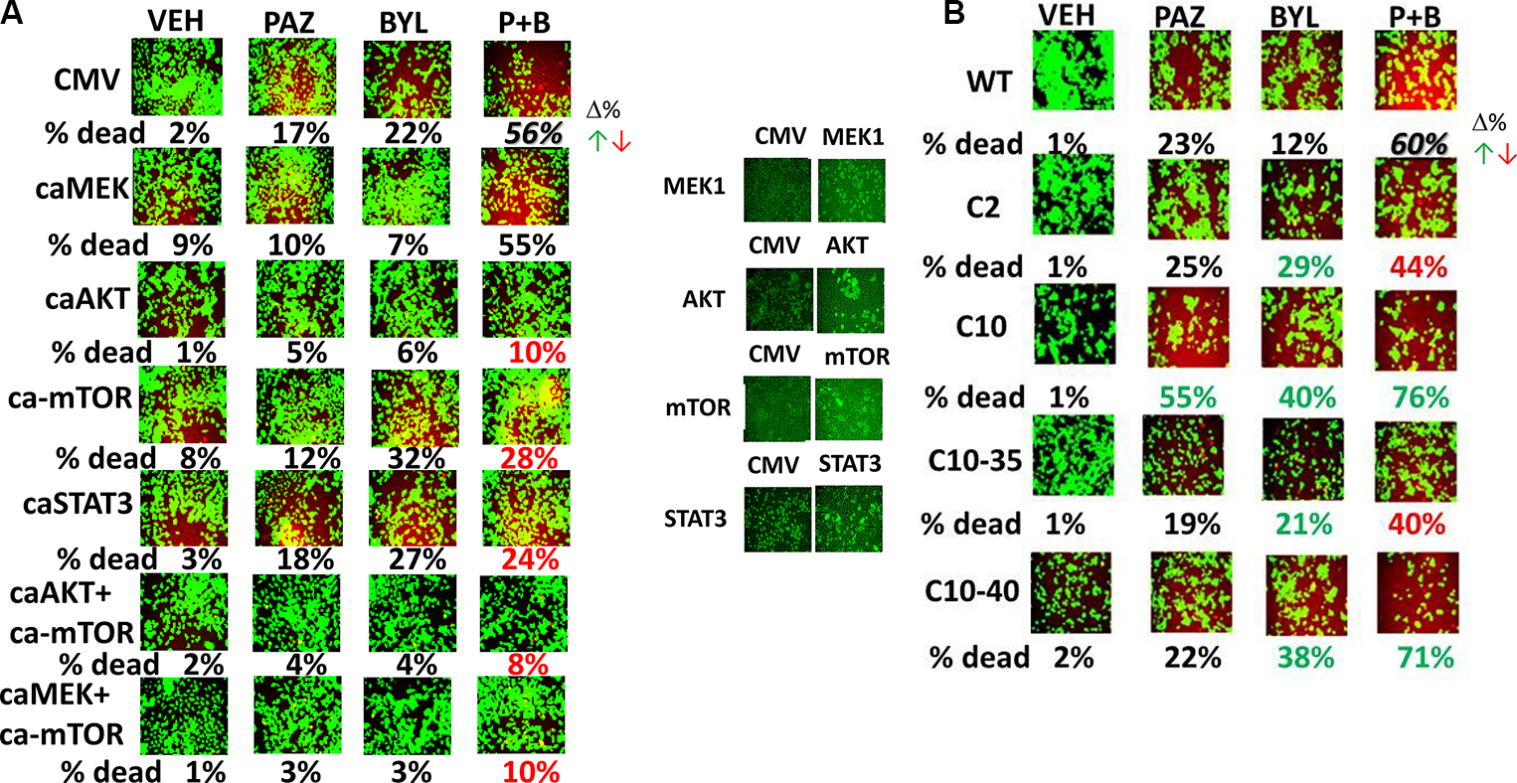
Elevated PI3K signaling predicts for tumor cell responsiveness to [pazopanib + BYL719] exposure (**A**)ADOR cells were transfected with an empty vector plasmid (CMV) or with plasmids to express: activated MEK1; activated AKT; activated mTOR; or activated STAT3. Twenty four h after transfection the cells were treated with vehicle control, pazopanib (2.0 μM), BYL719 (0.5 μM) or the drugs combined for 24 h after which cell viability was determined. Values in red or green represent statistically significant changes in the fluorescence level compared to vehicle control, *p* < 0.05, *n* = 3 +/− SEM. (**B**) HCT116 cells (wild type, expressing a single allele of K-RAS D13; C2 deleted for mutant K-RAS D13; C10, C2 deleted for mutant K-RAS D13 expressing H-RAS V12; C10–35, C2 deleted for mutant K-RAS D13 expressing H-RAS V12 S35 point mutant that selectively activates ERK1/2; C10–40, C2 deleted for mutant K-RAS D13 expressing H-RAS V12 C40 point mutant that selectively activates PI3K. Cells were treated with vehicle control, pazopanib (2.0 μM), BYL719 (0.5 μM) or the drugs combined for 24 h after which cell viability was determined. Values in red or green represent statistically significant changes in the fluorescence level compared to vehicle control, *p* < 0.05, *n* = 3 +/− SEM.

We then attempted to define the most important cyto-protective signal transduction pathways which act to counter [pazopanib + BYL719] lethality. Expression of constitutively active forms of AKT, mTOR or STAT3 significantly reduced the lethality of [pazopanib + BYL719] (Figure 12A). Expression of activated MEK1 was not protective, however activated MEK1 enhanced the ability of activated mTOR to protect the tumor cells. To further clarify the roles of signaling pathways downstream of mutated active RAS proteins, we made use of HCT116 colon cancer cells expressing different forms of mutated active K-RAS and H-RAS, previously published by our group [28, 29]. Pazopanib and BYL719 strongly interacted to kill wild type HCT116 cells that express a mutated active K-RAS protein (Figure 12B). In HCT116 C2 cells, that lack the single allele of mutated active K-RAS, a significant reduction in the lethality of [pazopanib + BYL719] was observed whereas the ability of BYL719 to kill these cells was significantly increased. In HCT116 C10 cells, that are the C2 cells stably transfected to express mutated active H-RAS V12, we found that the abilities of pazopanib, BYL719 and [pazopanib + BYL719] to kill were all significantly enhanced; this correlates to the very high levels of PI3K pathway activity we have previously observed in this cell line. In the HCT116 C10-35 line, that is the C2 line transfected to stably express an H-RAS V12 mutant that specifically activated the ERK1/2 pathway, the lethality of BYL719 was significantly enhanced but that of the drug combination significantly reduced. In the HCT116 C10-40 line, that is the C2 line transfected to stably express an H-RAS V12 mutant that specifically activated the PI3K pathway, the lethality of BYL719 and that of the drug combination significantly were significantly enhanced. Collectively the data in Figure 12 argues that pazopanib and BYL719 interact most strongly to kill in cells that have an activated PI3K-AKT-mTOR signaling pathway.

**Figure 13:**
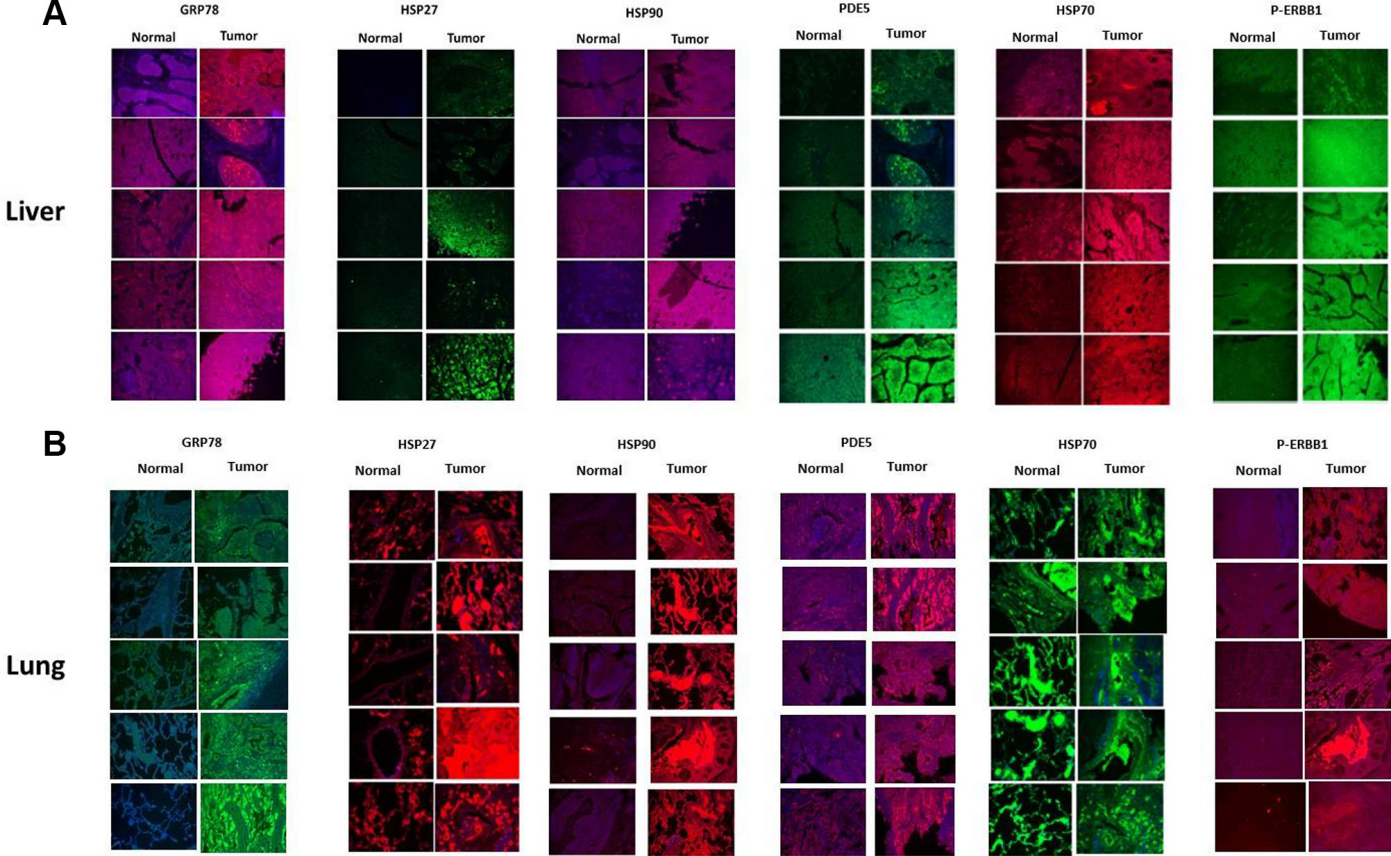
Liver and Lung tumor cells express higher levels of GRP78 and HSP27 than non-transformed cells from the same tissue from the same patient A liver tissue micro-array was purchased (http://www.novusbio.com/Liver-Tissue-Micro-Array_NBP2–30221.html) and antigen retrieval performed on the tissue sections. Immuno-histochemical staining was performed to determine and compare the expression of PDE5, HSP70, Phosopho-ERBB1, HSP90, GRP78 and HSP27 in normal liver alongside paired tumor tissue from the same patient.

Using hepatic tissue and lung tissue micro-arrays from a commercial vendor we compared the expression of HSP90, HSP70, GRP78 and HSP27 in cancer tissue and from matched normal tissue from the same patient. Liver and Lung cancer cells strongly over-expressed GRP78 and HSP27 compared to normal tissues, arguing that these chaperones play a greater role in the biology of tumor cells than non-transformed cells, and represent a therapeutic window of opportunity in these diseases (Figures 13A and 13B). Liver and lung tumor cells also expressed more phosphodiesterase 5 and phosphorylated ERBB1 than matched normal tissues.

**Figure 14:**
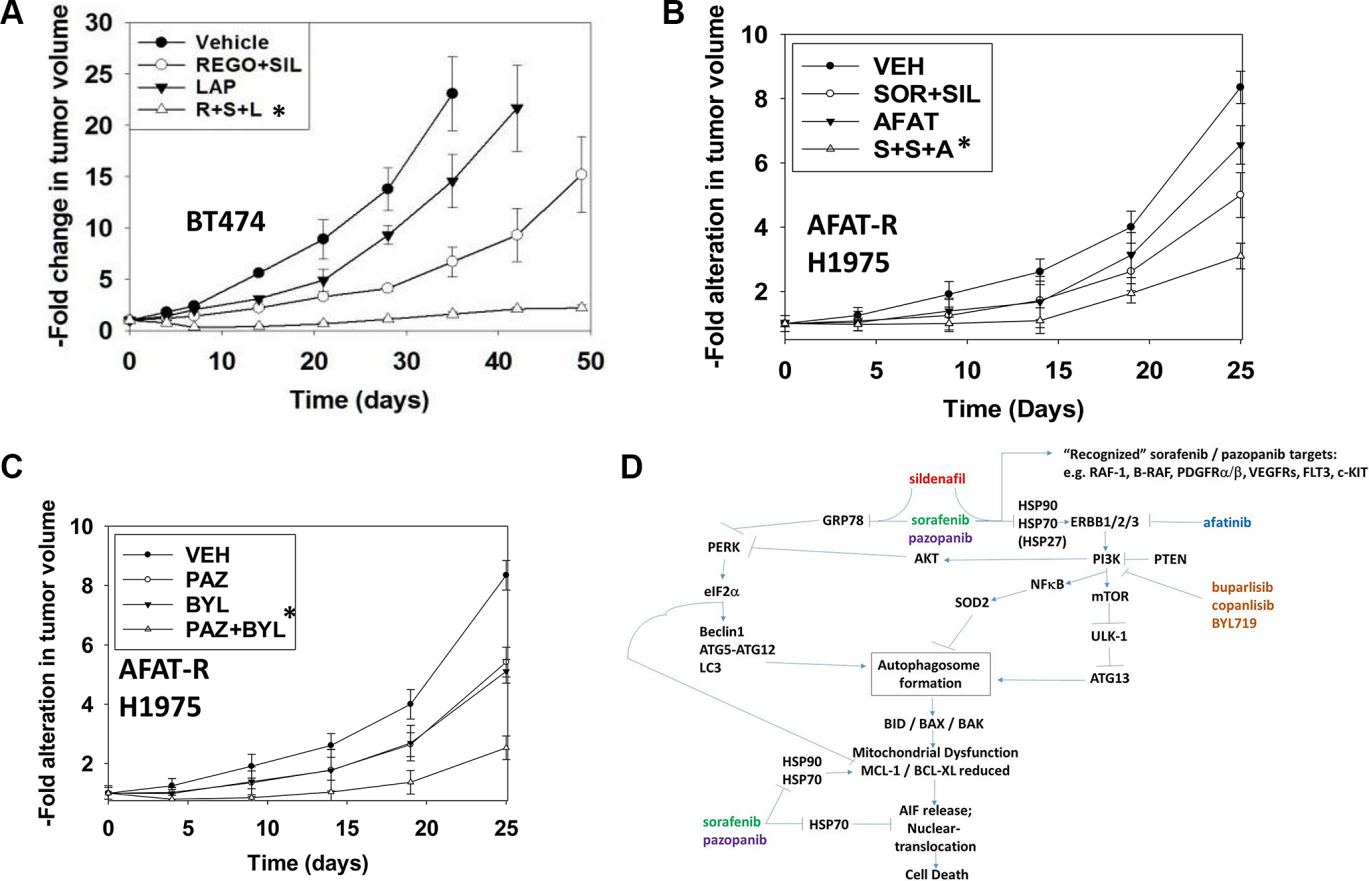
ERBB1/2 inhibitors and PI3K inhibitors enhance the lethality of [sorafenib/pazopanib/regorafenib + sildenafil] in mammary carcinoma cells (**A**) BT474 cells (1 × 10^7^) were injected into the 4^th^ mammary fat pad of athymic mice and tumors permitted to form until their volume was ~50 mm^3^. Mice were treated PO for *4 days* with: vehicle; [regorafenib (25 mg/kg QD) + sildenafil (10 mg/kg BID)]; lapatinib (50 mg/kg BID); or the three drugs together. Tumor volumes were measured on the days indicated in the graph (*n* = 10 mice per condition +/− SEM). (**B** and **C**) Afatinib resistant H1975 cells (1 × 10^7^) were injected into the rear flank of beige SCID mice and tumors permitted to form until their volume was ~15 mm^3^. Mice were treated PO for *4 days* with: vehicle; [sorafenib/pazopanib (25 mg/kg BID) + sildenafil (10 mg/kg BID)]; afatinib (25 mg/kg QD); BYL719 (25 mg/kg QD); or the drugs together in combination as indicated. Tumor volumes were measured on the days indicated in the graph (*n* = 10 mice per condition +/− SEM). Statistical examination of *in vivo* animal survival data utilized log rank statistical analyses between the different treatment groups. Differences with a *p*-value of < 0.05 were considered to be statistically significant. (**D**) Sorafenib can act as a weak inhibitor of chaperone ATPase inhibitor and it dysregulates HSP90 and HSP70 chaperones as well as altering the biology of small HSPs such as HSP27. This “sets the scene” for reduced signaling into the PI3K-(AKT)-mTOR pathway and increased signaling by PERK-eIF2α pathway that collectively through increased ATG13 S318 phosphorylation and elevated Beclin1 expression promote a toxic form of autophagy. Downstream of autolysosomes and the release of cathepsin and calpain enzymes into the cytosol is their cleavage of BID and with reduced expression of mitochondrial protective proteins leads to activation of BAX and BAK and the release of AIF into the cytosol. Because HSP70 function has been reduced AIF is more capable of translocating from the cytosol into the nucleus where it executes the tumor cell.

Finally, we attempted to validate our laboratory based studies in animal model systems. We initially chose BT474 mammary tumor cells for our studies, freshly purchased from the ATCC. The BT474 line expresses a mutant p53 protein and over-expresses the plasma membrane receptor ERBB2 (HER2). In agreement with our prior *in vitro* data combining [sorafenib + sildenafil + afatinib], and its effect on chaperone activity and total ERBB2 expression, *in vivo* in athymic mice the three drug combination of [regorafenib + sildenafil + lapatinib] profoundly suppressed BT474 mammary tumor growth to a significantly greater extent than the two drug [regorafenib + sildenafil] combination (Figure 14A, *p* < 0.05) [9]. We next grew afatinib resistant H1975 tumors in the rear flanks of beige SCID mice and determined the response of these cells *in vivo* to [sorafenib + sildenafil + afatinib] and to [pazopanib + BYL719]. Afatinib enhanced the anti-tumor effects of [sorafenib + sildenafil] and [pazopanib + BYL719] combined were significantly more effective at suppressing tumor growth than either agent individually (Figures 14B and 14C, both *p < 0.05).

## DISCUSSION

At present the drug combinations of [regorafenib + sildenafil] and [sorafenib + sildenafil + valproate] are phase I and phase II clinical trials at Massey Cancer Center (NCT02466802, NCT01817751), respectively. From our prior studies in Tavallai et al and Roberts et al we also were aware that tumors treated with [sorafenib/regorafenib + sildenafil] re-grow after therapy with higher ERBB1 phosphorylation and that the combination of [sorafenib/regorafenib + sildenafil] can reduce the detection by in-cell western/immuno-fluorescence of the chaperones HSP90, GRP78 and HSP70 [6, 8]. The “big-picture” conclusions we had previously made concerning the biology of the [sorafenib/regorafenib + sildenafil] drug combination prior to the present manuscript were that the combination kills tumor cells selectively over non-transformed cells and does so by primarily inactivating and down-regulating anti-apoptotic protein expression together with those of chaperones which causes both death receptor activation and mitochondrial dysfunction as well as a prolonged intense endoplasmic reticulum stress response leading to high levels of autophagy flux and an apparent necroptotic form of tumor cell death.

Previously we have shown that [sorafenib/regorafenib/pazopanib + sildenafil] reduces expression of cytoplasmic as well as cell surface GRP78; and cell surface GRP78 had been shown to play an intimate role in maintaining PI3K/PDK-1/AKT activity [see bibliography in ref. 9]. The drug combinations also mediate reductions in HSP27 expression and changes in its localization, including in autophagosomes; and this further strengthens the mechanism for reducing PI3K pathway activity as several groups have shown that HSP27 plays a key chaperone function in facilitating PI3K/AKT activation, which in turn provides a potent downstream anti-apoptotic signal in its own right, and through mTOR an anti-autophagy signal. That [sorafenib/regorafenib/pazopanib + sildenafil] targets multiple chaperone proteins whose clients play essential roles in maintaining anti-apoptotic signaling through the PI3K pathway strongly supports the use of this combination at the very least as a cancer therapeutic index modulator to enhance the effectiveness of standard of care therapies, and in the case of our data, glioblastoma multiforme and non-small cell lung cancer.

The role of HSP27 in the regulation of other PI3K pathway regulated processes also connects our prior studies with [sorafenib/regorafenib + sildenafil] to the new data in this manuscript [6, 8]. As GRP78 and HSP27 both act to maintain PI3K/AKT signaling they also could be thought to very likely play a key role in the regulation of the PI3K-regulated kinase mTOR. And multiple studies by others have linked sorafenib/regorafenib to the regulation of mTOR, ER stress responses and autophagosome formation. Our present data strongly argues that regulation of mTOR function by HSP27 may be of greater importance from a therapeutic standpoint than modulation of AKT. Under conditions of nutrient surplus or growth factor stimulation mTOR is active and both enhances cell proliferation and mobility but also suppresses catabolic processes such as macro-autophagy through inhibitory phosphorylation of ULK- 1 (known as ATG-1 in yeast). We noted that the drug combination reduced the phosphorylation of mTOR at S2448, to a much greater extent than that of AKT T308, indicative of reduced mTOR kinase activity, which correlated with reduced phosphorylation of the inhibitory mTOR phosphorylation site in ULK-1, Serine 757. ULK-1 activation concomitant with Serine 757 dephosphorylation was demonstrated by increased ATG13 phosphorylation at Serine 318, a site known to be targeted by ULK-1. Thus combined loss of GRP78 and HSP27 facilitates a reduction in mTOR activity which in turn promotes ULK-1 –dependent ATG13 phosphorylation and hence autophagosome formation.

In previous work we had linked [sorafenib/regorafenib/pazopanib + sildenafil] effects at increasing autophagosome formation to reduced GRP78 expression and in parallel the activation of PERK and elevated endoplasmic reticulum stress signaling [6–9]. In transformed rodent fibroblasts we have previously shown that the expression of Beclin1 and LC3 can be regulated in a PERK dependent manner, in general agreement with the present data in human tumor cells showing that dominant negative eIF2 alpha also suppressed [sorafenib/regorafenib + sildenafil] –induced expression of the autophagy regulatory, and autophagy facilitating proteins, Beclin1 and LC3. Furthermore, in this manuscript we demonstrated that [sorafenib/regorafenib + sildenafil] causes the co-localization of both GRP78 and HSP27 with LC3 in punctate bodies i.e. autophagosomes. Thus collectively our data from previous work and the present studies argue that by modulating the function of the chaperones GRP78 and HSP27, regardless of the many other chaperones we also now know are effected by [sorafenib/regorafenib + sildenafil] exposure; it promotes toxic autophagosome formation through two distinct mechanisms and thus this is why the catabolic process is associated with [sorafenib/regorafenib + sildenafil] –induced tumor cell death rather than survival.

One of the key observations made in this manuscript and in the recent Booth et al manuscript that examines the biology of the afatinib resistant H1975 cells in greater detail is that afatinib resistant H1975 clones do not exhibit any additional “hotspot” mutations in proto-oncogenes compared to controls that would have been a priori predicted [10]. In the ADOR PDX isolate, again to our surprise, no well described proto-oncogene driving mutation was identified. Thus in both instances, we had to ask ourselves what “changes” in cell biology made the afatinib resistant H1975 cell clones resistant to afatinib and what “made” the ADOR cell isolate cancerous? The change in afatinib sensitivity cannot be simply ascribed to DNA mutations, as would be routinely analyzed in for each patient using the in vogue “personalized medicine” techniques. In contrast to studies examining nucleic acids, our analyses of protein expression and protein phosphorylation were much more informative as to how and why the H1975 cells became afatinib resistant and as to how and why ADOR cells are cancerous. The studies with the ADOR cells are a prime example of what information can be gleaned from tumor material after surgical removal of the tumor but before the patient has had their first visit with their medical-oncologist.

In the five weeks between surgery and the first meeting of the patient with their medical oncologist we were able in the Dent laboratory to define the expression and phosphorylation of multiple proteins whose impact on tumorigenesis in NSCLC would be a priori predicted to play a role in the biology of this patient specific tumor isolate. Within those five weeks we had discovered that ERBB3-PI3K-NFκB signaling, but not mutated active ERBB1 or mutated active K-/N-RAS, played a major driving role in ADOR cell growth/viability. We knew that the high stoichiometric phosphorylation of wild type PTEN at serine 380 in ADOR cells was one key additional player in permitting the ERBB3-*PI3K*-NFκB signal to progress down the signaling pathway. Yet, contrary to our expectation in a cell lacking a mutant active ERBB1, we also knew that the ERBB1/2/4 inhibitors lapatinib and afatinib profoundly altered the biology of the ADOR cells as single agents, slowing tumor growth and radically altering the morphology and invasive potential of the cells. Whether such information, under the current medical and ethical guidelines, would have permitted the attending medical-oncologist to modify the standard of care for this patient (pemetrexed – platinum therapy) so that a better response could be specifically engineered for this patient is open to conjecture.

In conclusion, in this manuscript we have demonstrated that [sorafenib + sildenafil] and [pazopanib + sildenafil] lethality can be enhanced by inhibition of ERBB1/2/4-PI3K-NFκB signaling and that both ER stress signaling and inactivation of mTOR plays key roles in this process. The schematic in Figure 14D attempts to describe the multiple overlapping mechanisms by which [sorafenib/pazopanib + sildenafil +/− afatinib or +/− PI3K inhibitor] interact to kill tumor cells. Sorafenib/pazopanib inhibit the activities of multiple serine/threonine/tyrosine kinases as well as the chaperones GRP78, HSP90 and HSP70 (and other chaperones, e.g. HSP60, data not shown). Loss of GRP78/HSP90/HSP70 chaperone functions reduces gross protein translation through elevated ER stress eIF2α signaling leading to lower expression of growth factor receptors such as ERBB1-4 and mitochondrial protective proteins such as MCL-1, but also increases expression of the autophagosome regulatory proteins Beclin1, ATG5 and LC3. Sorafenib/pazopanib, likely through HSP70 dysregulation, causes inactivation of the chaperone HSP27, which together with loss of GRP78, results in a destabilized PI3K signaling pathway, with lower levels of AKT and mTOR activity. Reduced AKT signaling de-represses the ER stress kinase PERK further facilitating the ER stress response caused by loss of GRP78 function. Reduced mTOR activity results in the dephosphorylation of ULK-1 at serine 757, which leads to activation of ULK- 1 kinase activity with the phosphorylation of ATG13, the gate-keeper protein for autophagosome formation, being elevated. Increased autophagosome/autolysosome flux leads to the release of cathepsins and calpains into the cytosol where they catalyze the cleavage of the toxic BH3 domain protein BID that occurs concomitantly with the ER stress –induced decline in MCL-1 and BCL-XL expression; and, loss of HSP90 and HSP70 function also enables the reduction in expression of these tumor cell protective proteins. The alterations in BID/MCL-1/BCL-XL function result in the activation of BAX and BAK, and the release of apoptosis inducing factor into the cytosol. Additionally, because the function of HSP70 has been suppressed, AIF more readily translocates from the cytosol to the nucleus where it executes the tumor cell.

## MATERIALS AND METHODS

### Materials

Sildenafil, sorafenib tosylate, lapatinib, afatinib, BKM120 (buparlisib), BYL718, copanlisib, pazopanib and regorafenib were purchased from Selleckchem (Houston, TX). Trypsin-EDTA, DMEM, RPMI, penicillin-streptomycin were purchased from GIBCOBRL (GIBCOBRL Life Technologies, Grand Island, NY). Cells were purchased from the ATCC and were not further validated beyond that claimed by ATCC. Cells were re-purchased every ~6 months. Primary human GBM cells, developed by Dr. C.D. James when at the Mayo Clinic (Rochester, MN) has been described previously. ADOR non-small cell lung cancer cells and OSRC-1 osteo-sarcoma cells are personal donations from the patients to the Dent laboratory. De novo cisplatin resistant PDX “Spiky” ovarian cancer cells were very kindly provided by Dr. Karen Paz (Champions Oncology, NJ). The plasmid to express GRP78/BiP/HSPA5 was kindly provided to the Dent laboratory by Dr. A.S. Lee (University of Southern California Los Angeles, CA). The plasmids to express HSP90, HSP70, HSP27, active STAT3, active mTOR, dom. neg. IκB S32A S36A, eIF2α S51A, and all others listed in this manuscript were purchased from Addgene (Cambridge, MA) (Figure 12A; Figure S1A). Commercially available validated short hairpin RNA molecules to knock down RNA/protein levels were from Qiagen (Valencia, CA) or were supplied by collaborators [1–12, 28, 29] (Figure 12A; Figure S1A).

### Methods

#### Culture and *in vitro* exposure of cells to drugs

All cell lines were cultured at 37°C (5% (v/v CO_2_) *in vitro* using RPMI supplemented with dialyzed 5% (v/v) fetal calf serum and 10% (v/v) Non-essential amino acids. *In vitro* drug treatments were from 100 mM stock solutions of each drug and the maximal concentration of Vehicle (DMSO) in media was 0.02% (v/v). Cells were not cultured in reduced serum media during any study in this manuscript.

### Transfection of cells with siRNA or with plasmids

#### For plasmids

Cells were plated and 24 h after plating, transfected. Plasmids expressing a specific mRNA (or siRNA) or appropriate vector control plasmid DNA was diluted in 50μl serum-free and antibiotic-free medium (1 portion for each sample). Concurrently, 2 μl Lipofectamine 2000 (Invitrogen), was diluted into 50 μl of serum-free and antibiotic-free medium (1 portion for each sample). Diluted DNA was added to the diluted Lipofectamine 2000 for each sample and incubated at room temperature for 30 min. This mixture was added to each well/dish of cells containing 200μl serum-free and antibiotic-free medium for a total volume of 300 μl, and the cells were incubated for 4 h at 37°C. An equal volume of 2x medium was then added to each well. Cells were incubated for 24 h, then treated with drugs.

#### Transfection for siRNA

Cells from a fresh culture growing in log phase as described above, and 24 h after plating transfected. Prior to transfection, the medium was aspirated and serum-free medium was added to each plate. For transfection, 10 nM of the annealed siRNA, the positive sense control doubled stranded siRNA targeting GAPDH or the negative control (a “scrambled” sequence with no significant homology to any known gene sequences from mouse, rat or human cell lines) were used. Ten nM siRNA (scrambled or experimental) was diluted in serum-free media. Four μl Hiperfect (Qiagen) was added to this mixture and the solution was mixed by pipetting up and down several times. This solution was incubated at room temp for 10 min, then added drop-wise to each dish. The medium in each dish was swirled gently to mix, then incubated at 37°C for 2 h. Serum-containing medium was added to each plate, and cells were incubated at 37°C for 24 h before then treated with drugs (0–24 h). Additional immuno-fluorescence/live-dead analyses were performed at the indicated time points.

### Animal studies (lung cancer)

Animal studies were performed according to Federal Law and under an approved Virginia Commonwealth University IACUC protocol (#AD10001065). For studies to generate afatinib resistant H1975 cells, athymic nude mice (~20 g) were injected with 1 × 10^7^ H1975 cells into their rear flank. Tumors were permitted to form for 7 days with tumors at that time exhibiting a mean volume of 25–50 mm^3^. Athymic mice were treated by oral gavage twice every day BID for four days with vehicle (Cremophore) or with afatinib (50 mg/kg). After cessation of drug treatment tumors treated twice daily with afatinib showed a reduction in tumor volume of all treated tumors to 0 mm^3^ for approximately 7 days after which tumors began to slowly re-grow. Recurrent tumors were isolated twenty five days after they exhibited the initial re-formation of a small tumor, when they had a volume of ~500 mm^3^, portions were either snap-frozen or were digested to release individual tumor cells, and cells from each tumor clone maintained separately i.e. we generated 5 control/vehicle treated clones from 5 separate tumors and we generated 5 afatinib-resistant clones from 5 separate tumors. Control treated tumors were also isolated when they had a volume of ~500 mm^3^. Of significant note for clonal characterization, the isolated afatinib treated tumor clones’ cells were only growth inhibited by afatinib when cultured *in vitro* with daily supplementation at concentrations >> 2 μM, and as such these afatinib resistant cells were routinely passaged in a pulsatile fashion between experiments in growth media containing only 1 μM afatinib to maintain the afatinib resistant phenotype but not to promote further selective pressure on drug resistance and thus also did not cause selection of surviving clones due to non-ERBB1/2/4 off-target effects [10].

### Treatment of BT474 tumors with drugs

Animal studies were performed according to Federal Law and under an approved Virginia Commonwealth University IACUC protocol (#AD10001065). Athymic female mice (~20 g) were used. BT474 cells (1 × 10^7^) were injected into the 4^th^ mammary fat pad and tumors permitted to form until their volume was ~50 mm^3^. Mice were treated PO for 5 days with: vehicle; [regorafenib (25 mg/kg QD) + sildenafil (10 mg/kg BID)]; lapatinib (50 mg/kg BID); or the three drugs together. Tumor volumes were measured on the days indicated in the graph (*n* = 10 mice per condition +/− SEM).

### Treatment of afatinib resistant H1975 tumors with drugs

Animal studies were performed according to Federal Law and under an approved Virginia Commonwealth University IACUC protocol (#AD10001065). Afatinib resistant H1975 cells (1 × 10^7^) were injected into the rear flank of SCID mice and tumors permitted to form until their volume was ~50 mm^3^. Mice were treated PO for 4 days with: vehicle; [sorafenib/pazopanib (25 mg/kg BID) + sildenafil (10 mg/kg BID)]; afatinib (25 mg/kg QD); BYL719 (25 mg/kg QD); or the drugs together in combination as indicated. Tumor volumes were measured on the days indicated in the graph (*n* = 10 mice per condition +/− SEM).

### Detection of cell viability, protein expression and protein phosphorylation by immuno-fluorescence using a Hermes WiScan machine

The technology that permitted the Dent laboratory to perform so many experiments so rapidly to provide this protein biochemistry form of personalized medicine is a wide-field microscope with a motorized stage and electronic focusing that permits the use of 96 well plates for study: the Hermes WiScan microscope from IDEA Bio-systems (http://www.idea-bio.com/). The system in the present studies has been used set at 10X magnification, in essence a form of “in-cell western blot;” and set at 60X for fine detail examination of the cells and co-localization studies. The company claims that the automated Hermes machine can provide high image quality with rapid image acquisition speed. In practice, the Dent laboratory experience confirms this claim. Because a 96 well plate can be used in the system, hypothetically, a single experiment in triplicate could have 32 separate treatment conditions. Cells (4 × 10^3^) are plated into each well of a 96 well plate, and cells permitted to attach and grow for the next 18 h. Based on the experiment, after 18 h, cells are then either genetically manipulated, or are treated with drugs. For genetic manipulation, cells are transfected with plasmids or siRNA molecules and incubated for an additional 24 h. Cells are treated with vehicle control or with drugs at the indicated final concentrations, alone or in combination. Cells are then isolated for processing at various times following drug exposure. The 96 well plate is centrifuged/cyto-spun to associate dead cells (for live-dead assays) with the base of each well. For live dead assays, after centrifugation, the media is removed and cells treated with live-dead reagent (Thermo Fisher Scientific, Waltham MA) and after 10 min this is removed and the cells in each well are visualized in the Hermes instrument at 10X magnification. Green cells = viable; yellow/red cells = dying/dead. The numbers of viable and dead cells were counted manually from three images taken from each well combined with data from another two wells of separately treated cells (i.e. the data is the mean cell dead from 9 data points from three separate exposures, approximately 200 cells). For immuno-fluorescence studies, after centrifugation, the media is removed and cells are fixed in place and permeabilized using ice cold PBS containing 0.4% paraformaldehyde and 0.5% Triton X-100. After 30 min the cells are washed three times with ice cold PBS and cells are pre-blocked with rat serum for 3 h. Cells are then incubated with a primary antibody to detect the expression/phosphorylation of a protein (usually at 1:100 dilution from a commercial vendor) overnight at 37°C. Cells are washed three times with PBS followed by application of the secondary antibody containing an associated fluorescent red or green chemical tag. After 3 h of incubation the antibody is removed and the cells washed again. The cells are visualized at either 10X or 60X in the Hermes machine for imaging assessments. Using Hermes system software, the intensity of the fluorescent signal in multiple images from each condition is determined, and included with the images, as indicated. All immunofluorescent images for each individual protein/phospho-protein are then taken using the identical machine settings so that the levels of signal in each image can be directly compared to the level of signal in the cells treated with drugs. Similarly, for presentation, the enhancement of image brightness/contrast using PhotoShop CS6 is simultaneously performed for each individual collected set of protein/phospho-protein pictures to permit direct visual comparison of the image intensity between treatments. Antibodies used include: HSP90 (E289) (Cell Signaling); HSP90 (#2928) (Abcam); HSP90 (ab195575) Abcam; HSP90 3G3 (13495) (Abcam); GRP78 (50b12) (31772) (Cell Signaling); GRP78 (ab191023) Abcam; GRP78 (ab103336) Abcam; GRP78 (N-20) (sc-1050) Santa Cruz; HSP27 (G31) (2402P) Cell Signaling); HSP27 [EP1724Y] (ab62339) Abcam; HSP27 (H-77) (sc-9012) Santa Cruz; HSP27 (LS-C31836) Lifespan science Corp.

### Tissue microarray and immunostaining

The human HCC and NSCLC tissue microarrays were purchased from Imgenex Corp. Antigen retrieval was performed by the Department of Pathology, VCU with antibody staining in the Dent lab.

### Isolation of GST-NH2 terminal HSP90 from bacteria and the measurement of its ATPase activity

*E. coli* BL21 [F–, *ompT*, *hsdS* (rB, mB), *gal*] were transformed with a plasmid to express a fusion protein of the NH2-terminal portion of HSP90 fused to glutathione-S-transferase (GST) (purchased from Addgene). Bacteria were grown at 24°C with shaking until the A600 reached 0.6. IPTG was added to a final concentration of 0.1 mM and the incubation continued for an additional 6 hours. Bacteria were recovered by centrifugation and stored on ice. The cell pellet was resuspended in 50 μl of ice-cold 1X PBS per ml of culture. The re-suspended cells were mechanically disrupted using a probe sonicator, on ice, in short 5 second bursts. Triton X-100 was added to a final concentration of 1% (v/v) followed by 30 minutes of gentle shaking to aid in solubilization of the fusion protein. Bacterial debris and denatured proteins were removed by centrifugation at 15,000 × g for 20 min at 4°C. The supernatant was removed and immediately mixed with pre-equilibrated Glutathione Sepharose 4B (2 ml of a 50% Sepharose slurry is mixed with 100 ml of clarified bacterial sonicate. The Sepharose slurry is gently rotated in a cold room for 30 min. The slurry mixture is centrifuged (10,000 x g for 10 min) and the supernatant discarded. The Glutathione Sepharose 4B is washed with 10 bead volumes of 1X PBS. The Sepharose slurry is centrifuged (5,000 x g, 5 min), and the supernatant discarded. The Sepharose beads are washed three times. Chaperone ATPase activity using the ATPlite 1step kit (PerkinElmer) was determined using GST-NH_2_ terminal HSP90 still linked to the Glutathione Sepharose 4B beads. The Sepharose beads are equilibrated in the reaction buffer provided by the manufacturer for 30 min with gentle mixing, and the beads recovered by centrifugation. The beads are then resuspended 1:1 with reaction buffer. To each well of a 96 well plate is added 50 μl of bead slurry and 50 μl of substrate buffer solution containing vehicle control or drug to achieve the desired final concentration. The reactions are started using a multi-channel pipette delivering 50 μl of reconstituted reagent to each well. The plate is placed in foil in an orbital shaker at 37°C for 15 min. The plate is removed; centrifuged to remove floating Sepharose beads; and 100 μl of the supernatant from each well placed into a new well in another 96 well plate. The light emitted from each well/treatment condition is quantified using a Vector 3 plate reader (*n* = 3 of three studies +/− SEM).

### Molecular dynamics

The model system for HSP90 was built using VMD. For the model, the coordinates of human HSP90 in complex with ACP (PDB refcode 3T10) were used. The protein was inserted into a cubic water box of size 80 × 80 × 80 Å^3^. The whole system consisted of 47971 atoms. For MD simulations, NAMD 2.10 was used. Ligand and protein were restrained to their original positions and the system was equilibrated at constant temperature and pressure (300 K, 1 atm, NPT ensemble) for 0.5 ns. Simulations were done using the CHARMM36 force-field in combination with the CGenFF forcefield where needed. Then 5 ns of production MD was performed. Docking (Autodock Vina) was done against conformations extracted from this run. Initial CGenFF-compatible parameters for the pazopanib were derived using the CGenFF website and subsequently optimized. Subsequently, 50 ns of accelerated MD with Generalized Born Implicit Solvent (GBIS) for both HSP90 in complex with pazopanib and apo-HSP90 was performed.

### Data analysis

Comparison of the effects of various treatments was performed using one way analysis of variance followed by a two tailed Student's *t*-test. Statistical examination of *in vivo* animal survival data utilized log rank statistical analyses between the different treatment groups. Differences with a *p*-value of < 0.05 were considered to be statistically significant. Experiments shown are the means of multiple individual data points from multiple separate experiments (± SEM).

## SUPPLEMENTARY MATERIALS FIGURES



## Abbreviations

CD: cluster of differentiation
PDGF: platelet derived growth factor
EGF: epidermal growth factor
EGF-R, HER-1, ErbB1, or ErbB: epidermal growth factor receptor
SIL: sildenafil also called Viagra
TAD: tadalafil also called Cialis
PTEN: Phosphatase and tensin homolog
R: receptor
dn: dominant negative
CMV: empty vector control plasmid
COX: cyclooxygenase
P: phospho-
ca: constitutively active
WT: wild type
PERK: PKR like endoplasmic reticulum kinase
HSP: heat shock protein
GRP: glucose regulated protein
