# Supplementary material for: Multi-kinase inhibitors interact with sildenafil and ERBB1/2/4 inhibitors to kill tumor cells in vitro and in vivo
Source: Oncotarget. 2016 May 31;7(26):40398–417. doi: 10.18632/oncotarget.9752 (PMC5130016; doi:10.18632/oncotarget.9752)
Supplement: Supplementary file 1 [file oncotarget-07-40398-s001.pdf]

Multi-kinase inhibitors interact with sildenafil and ERBB1/2/4 inhibitors to kill tumor cells *in vitro* and *in vivo*

Supplementary Materials

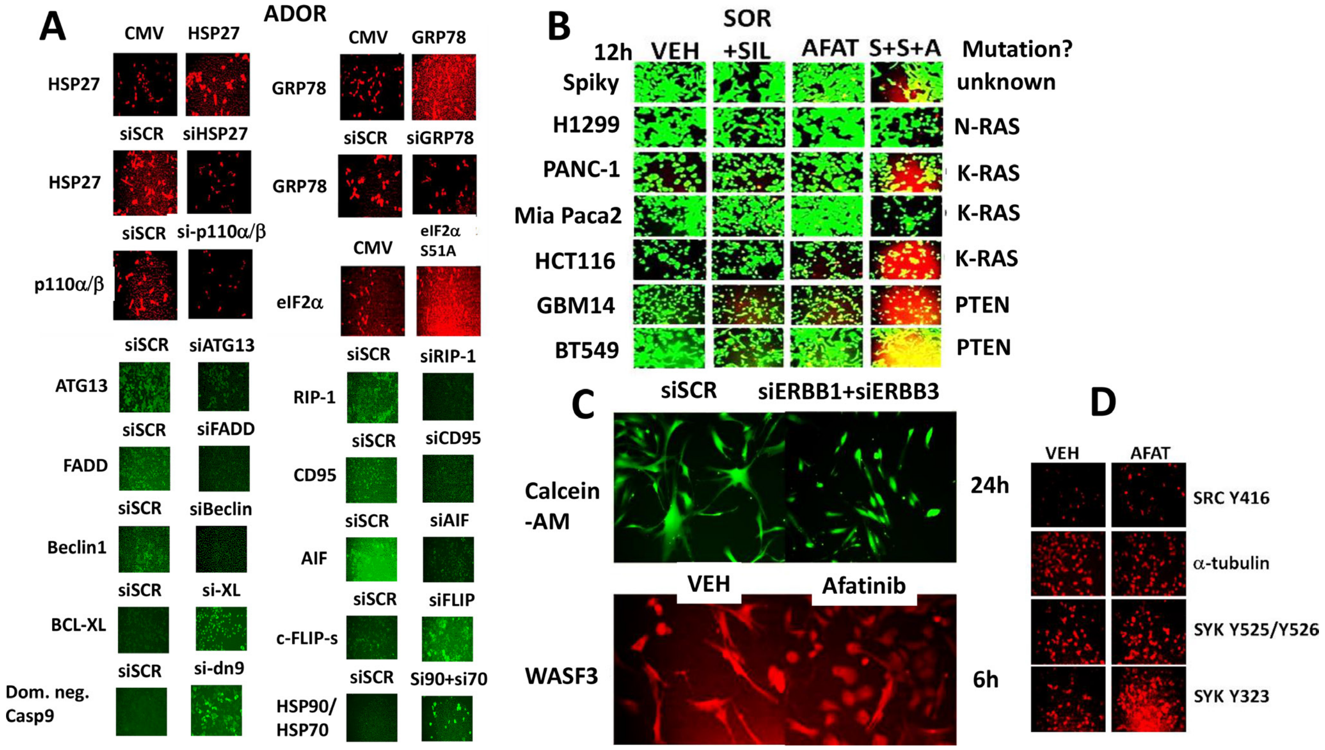

Supplementary Figure S1: ERBB1/3 signaling through PI3K controls cell morphology and cell viability. (**A**) Control knock down or over-expression immuno-fluorescence images of proteins studied in this manuscript. (**B**) Spiky a de novo highly resistant PDX ovarian tumor cell isolate; BT549, HCT116, GBM14, Mia Paca 2, PANC1 and H1299 cells were treated with vehicle control or [sorafenib (2.0 µM) + sildenafil (2 µM)] or afatinib (1 µM) or the three drugs in combination, for 24 h after which cell viability was determined (n = 3 +/– SEM). (**C**) ADOR cells were either transfected with scrambled siRNA or with siRNAs together to knock down ERBB1 and ERBB3, and imaged 24 h after transfection (upper, calcein-AM stained green); ADOR cells were treated with vehicle or afatinib (1 µM) for 6h after which cells were then fixed in place and permeabilized using 0.5% Triton ×100. Immuno-fluorescence was performed to detect the expression of WASF3 at 10× magnification (lower, red stain). (**D**) ADOR cells were treated with vehicle or afatinib (1 µM) for 6 h after which cells were then fixed in place and permeabilized using 0.5% Triton X100. Immuno-fluorescence was performed to detect the expression of alpha-tubulin and the phosphorylation of SRC Y416, SYK Y525/Y526 and SYK Y323 at 10× magnification.

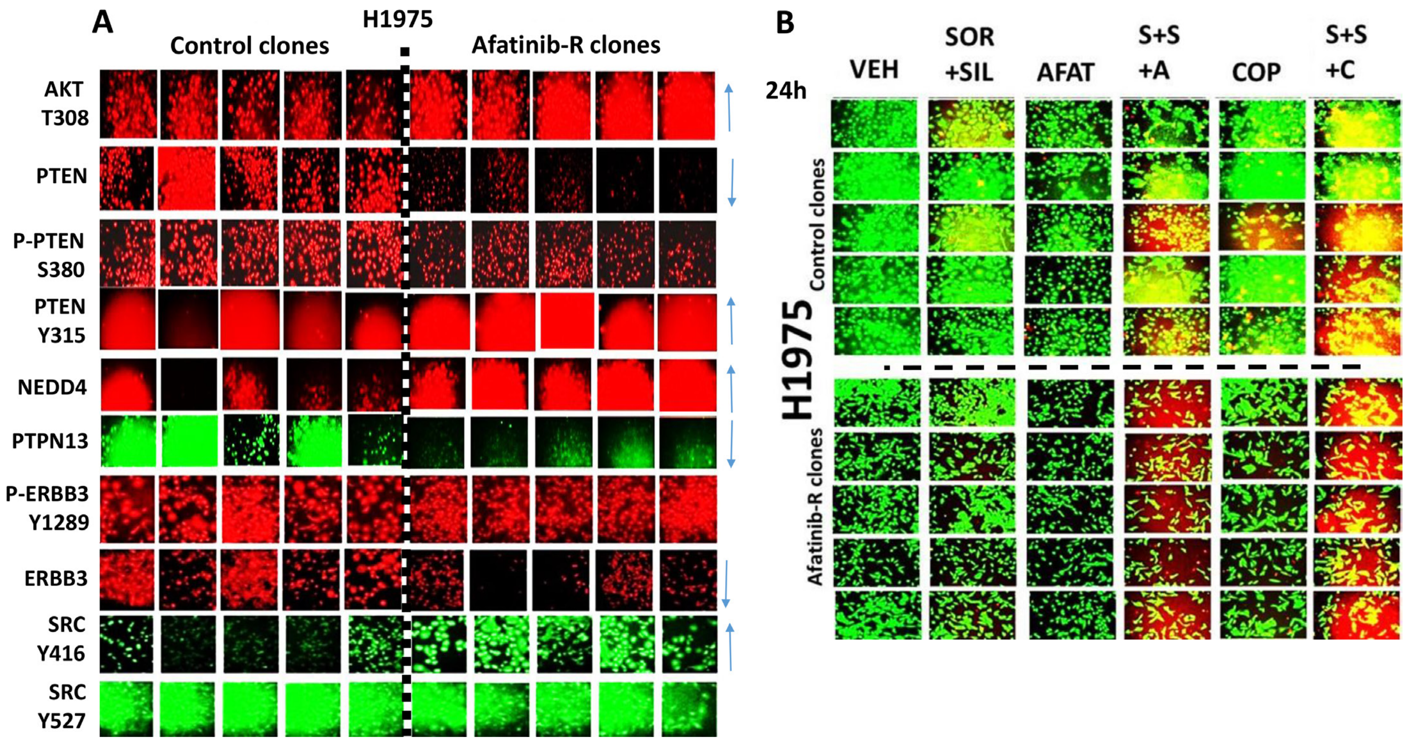

Supplementary Figure S2: In vivo generated afatinib resistant H1975 clones are more effectively killed by [sorafenib + sildenafil] than control clones. (**A**) Control and afatinib resistant H1975 clones, 24 h after plating, were fixed in place and permeabilized using 0.5% Triton X100. Immuno-fluorescence was performed to detect the phosphorylation levels and total expression of the indicated proteins. Images were at 10× magnification. Images were at 10× magnification. (**B**) Control and afatinib resistant H1975 non-small cell lung cancer clones were treated with vehicle control or with [sorafenib (2.0 µM) + sildenafil (2 µM)] +/– copanlisib (0.5 µM) or +/– afatinib (1.0 µM) for 12 h as indicated, after which cell viability was determined (n = 3 +/– SEM).
